# Digital twin brain simulator for real-time consciousness monitoring and virtual intervention using primate electrocorticogram data

**DOI:** 10.1038/s41746-025-01444-1

**Published:** 2025-02-10

**Authors:** Yuta Takahashi, Hayato Idei, Misako Komatsu, Jun Tani, Hiroaki Tomita, Yuichi Yamashita

**Affiliations:** 1 Department of Information Medicine, National Center of Neurology and Psychiatry, Tokyo, Japan; 2 Department of Psychiatry, Graduate School of Medicine, Tohoku University, Sendai, Japan; 3 Institution of Innovative Research, Tokyo Institute of Technology, Tokyo, Japan; 4 Cognitive Neurorobotics Research Unit, Okinawa Institute of Science and Technology, Okinawa, Japan

**Keywords:** Psychiatric disorders, Computational science, Network models

## Abstract

At the forefront of bridging computational brain modeling with personalized medicine, this study introduces a novel, real-time, electrocorticogram (ECoG) simulator, based on the digital twin brain concept. Utilizing advanced data assimilation techniques, specifically a Variational Bayesian Recurrent Neural Network model with hierarchical latent units, the simulator dynamically predicts ECoG signals reflecting real-time brain latent states. By assimilating broad ECoG signals from macaque monkeys across awake and anesthetized conditions, the model successfully updated its latent states in real-time, enhancing precision of ECoG signal simulations. Behind successful data assimilation, self-organization of latent states in the model was observed, reflecting brain states and individuality. This self-organization facilitated simulation of virtual drug administration and uncovered functional networks underlying changes in brain function during anesthesia. These results show that the proposed model can simulate brain signals in real-time with high accuracy and is also useful for revealing underlying information processing dynamics.

## Introduction

The concept of digital twins for human organs has recently emerged as a transformative technology in medicine. These twins use mathematical models to synchronize with real-time biological signals, enabling personalized treatment planning and pre-emptive treatment simulations^1–3^. Applications are advancing in areas such as cardiovascular treatment and pre-surgical planning^1,2^, with growing potential in neurological and psychiatric disorders, where disease heterogeneity highlights the need for personalized approaches^4^. While the brain’s complexity initially hindered digital twin development, advances in computational brain modeling, computational infrastructure, and brain activity monitoring techniques have recently enabled efforts to develop digital twin brains.

Technological contributions to digital twin brain development have included creation of brain function simulators at various scales. At the microscopic scale, models focus on neuronal components like ion channels and receptors, enabling simulation of pharmacological effects^5,6^. However, modeling the whole brain based on microscopic neuronal models incurs immense computational costs^6^. There is also the difficulty of non-invasively acquiring microscopic data from patient brains, making it hard to incorporate real-time patient information. Furthermore, models at the microscopic level are still not methodologically mature enough to handle phenomena associated with higher-level cognitive processes. In contrast, macroscopic models, such as reinforcement learning and Bayesian inference models, simplify brain-environment interactions into mathematical expressions, allowing modeling of cognitive processes^7,8^. However, the relationship between the limited number of variables that constitute these abstract models and the high-dimensional biological changes induced by therapeutic interventions, such as alterations in neural activity in various brain regions, remains unclear. Consequently, using this scale models for simulating therapeutic interventions still presents challenges.

In the context of bridging the gap between disparate scales of brain functionality, there has been growing interest in recent years in simulations of brain functions at the mesoscopic level^9–11^. Unlike simulations that focus solely on individual neurons or local neural circuits, this mesoscopic level simulator entails modeling neural activities and their interactive dynamics on a regional basis in the brain. At the mesoscopic level, techniques such as electroencephalography (EEG), electrocorticography (ECoG), magnetoencephalography (MEG), and functional magnetic resonance imaging (fMRI) enable real-time measurement of brain signals, providing dynamic insights into human brain function. It is also where we can apply neuromodulatory methods, such as transcranial direct current stimulation (tDCS) and transcranial magnetic stimulation (TMS), showing how closely connected this level of modeling is to advanced medical technologies. Furthermore, simulations at the whole-brain level are feasible at manageable computational cost^12^, making mesoscopic models promising candidates for digital twin brains.

However, research on digital twin brains using mesoscopic models is still in nascent stages, and has yet to fully realize requirements such as real-time synchronization and interventional simulations. In this endeavor, our aim is to endow digital twin brains with requisite capabilities through utilization of generative artificial neural network models at the mesoscopic level. Our current model’s distinctive feature lies in its utilization of data-driven end-to-end training, an approach that autonomously extracts information from observed brain signals to accurately simulate brain functions responsible for generating these signals. To further enhance the model’s capacity for real-time, accurate simulations, “data assimilation” is employed. This advanced technique supports data-driven optimization even after training is complete, thereby enabling the model to dynamically adapt to unknown and changing situations. However, the inherent complexity and noise in brain signals present a substantial challenge. To address this, we employ stochastic generative models with hierarchical latent states designed to capture intricate probabilistic structures underlying generation of these signals. This approach allows virtual therapeutic intervention to accurately simulate the process of generating changes in brain states and resulting brain signals.

In this study, we examine the utility of our proposed artificial neural network model as a digital twin brain, using ECoG data. Although ECoG is invasive, it enables measurement of high-frequency brain activity and provides significantly higher precision compared to traditional EEG^13,14^. Therefore, ECoG is particularly suitable for constructing a high-precision digital twin brain to capture latent brain activity. In this study, we employ data-driven training on ECoG signals to develop an ECoG generator (a digital twin brain prototype) and to evaluate its ability to meet the requirements of a digital twin, including real-time synchronization and intervention simulation, in the following way. First, we assessed whether the simulator could generate ECoG signals for test subjects in real time and with high accuracy using data assimilation techniques. This evaluation focused on two key points: whether the model’s latent states dynamically adjusted to reflect changes in the state of consciousness and whether generated ECoG signals were classified corresponding to the appropriate state of consciousness. Based on these findings, we further investigated the model’s capability to alter the state of consciousness in the generated virtual ECoG signals by manipulating its latent states. This involved conducting virtual intervention experiments at both the network level and the local brain region level to test whether generated signals reflected changes in consciousness corresponding to the interventions.

## Results

### System Overview

Development of the proposed digital twin brain simulator leveraged wide-area ECoG recordings of macaque monkeys, publicly accessible through the Neurotycho database^15,16^. These data consist of ECoG signals recorded from four macaques, both under anesthesia (ketamine and medetomidine) and in awake, resting condition with eyes closed. Of the 128 channels covering an entire hemisphere of the cerebral cortex, ECoG signals from 20 channels spanning 10 brain regions were selected (See Methods for details). ECoG data of all four individuals were utilized, with three individuals used for training and the remaining individual used for testing (data assimilation), employing a four-fold cross-validation methodology.

**Fig. 1:**
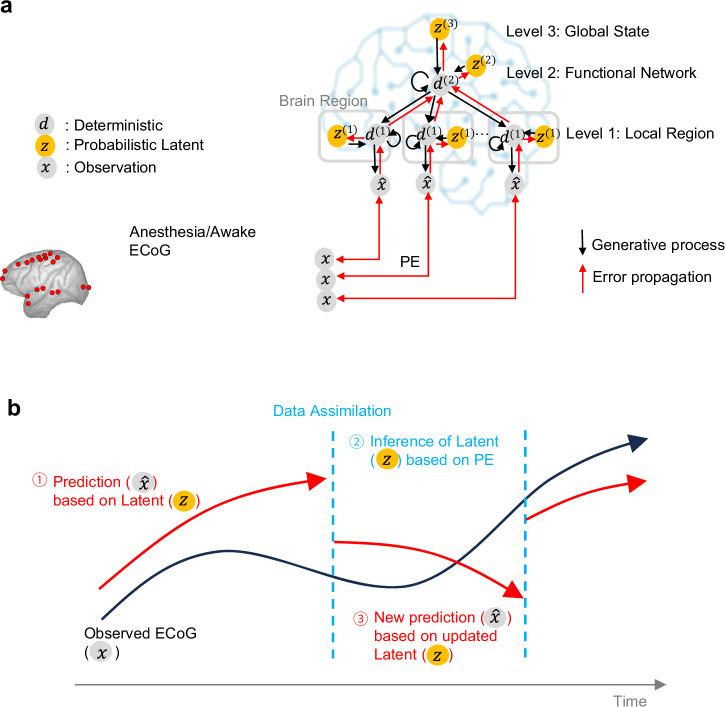
Neural Network Model and Data Assimilation Framework for electrocorticogram (ECoG) Signal Generation. **a** Model Overview. This model depicts the process by which ECoG signals are generated from latent states $z$ of the brain. The input $x$ consists of wide-area ECoG measurements from 20 channels across 10 brain regions in macaques. The latent variable $z$ possesses three hierarchical levels, each of which is stochastic. Latent states $z$, through synaptic connections of deterministic units $d$, lead to generation of ECoG signals $\hat{x}$. **b** Overview of the data assimilation procedure. For simplification, this figure does not show the time window. In an incrementally advancing window, predictions based on latent variables and updates of latent states based on prediction error (PE) are alternately repeated for a number of iterations.

The core component of the simulator is modeled using a Variational Bayesian Recurrent Neural Network (V-RNN), which is characterized by a temporal and spatial hierarchical structure (Fig. 1a)^17–20^. This structure comprises three levels: the local region level (the first level) operating at short time scales at the lowest layer, the functional network level (the second level) functioning at intermediate time scales, and the global state level (the third level) that operates over long time scales at the highest layer. These level-specific dynamics are realized by latent units z, which represent the stochastic latent state of each level, and deterministic units d, which convey time-scale-specific deterministic dynamics.

The V-RNN operates according to a dynamic generative model acquired through data-driven training (Fig. 1b), executing the following procedure: Initially, it estimates hierarchical and probabilistic latent states at time step $t$ (${z}_{t}$), as the “prior.” Based on this prior of ${z}_{t}$, the model predicts the ECoG signal ${\hat{x}}_{t}$. Subsequently, the actual observed ECoG signal ${x}_{t}$ is inputted. The prediction error is calculated in a time window that encompasses the past spanning $H$ time steps from $t$ ($t-H+1:t$). The latent state ${z}_{t-H+1:t}$ is updated based on this prediction error and is referred to as the posterior. Predictions are then made again based upon this posterior. The operation of making predictions and updating the posterior is repeated a predetermined number of times. Finally, the posterior is used to generate the prior at the next time step (${z}_{t+1}$) through the deterministic state ${d}_{t}$. This sequential repetition of updating the latent state based on predictive and observational data, a process designated as data assimilation, enables real-time estimation of latent states and generation of virtual ECoG.

### Real-time latent state estimation via data assimilation

As a result of this training, the proposed model successfully utilized acquired internal dynamics to generate 20-channel ECoG sequences during both anesthetized and awake resting states. Supplementary Fig. 1, which plots the loss throughout the training process, demonstrates a reduction in loss not only for training data, but also for validation data of test individuals. This indicates that the proposed model has acquired the capability to generalize, enabling it to generate ECoG sequences for test individuals. The similarity between observed ECoG signals and generated ECoG signals in the test set was further validated through comparisons of the power spectrum density (Supplementary Figs. 2 and 3) and the functional connectivity matrix (Supplementary Fig. 4). These findings collectively confirm the model’s ability to accurately replicate key features of the observed ECoG signals.

**Fig. 2:**
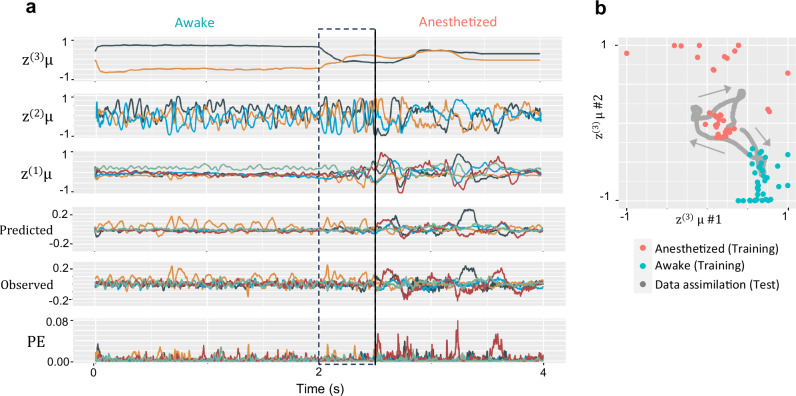
Real-time Prediction and Latent State Dynamics During Electrocorticogram (ECoG) Data Assimilation. **a** Prediction and latent state sequences in data assimilation. This figure illustrates the sequence of predictions and the mean (μ) of posterior distributions of latent states z^(1)^, z^(2)^, z^(3)^, generated in real-time in response to observed ECoG data. The Observed ECoG combines 2500 time steps (2.5 s) of awake ECoG observations followed by 1500 time steps (1.5 s) of anesthetized ECoG observations from a test individual (an individual not included in the training dataset). This figure plots values after data assimilation is complete across all time steps of the observed ECoG sequence. The vertical solid line shows the moment of transition between awake and anesthetized conditions in observed ECoG data, with dashed lines showing the time window range when reaching this moment. In this range, updates of latent states are made in response to the transition in the observed ECoG. Predicted and observed ECoG signals are visualized by selecting 5 channels from the original 20 channels for visual clarity. Specifically, these five channels are chosen from the frontal pole (FP), primary motor cortex (M1), primary somatosensory cortex (S1), intraparietal sulcus (IPS), and primary visual cortex (V1) regions. Similarly, the z^(1)^µ sequences were also visualized by selecting these five regions. **b** Example of the distribution of the mean (µ) of z^(3)^ obtained during training and data assimilation phases. In this study, the latent unit z^(3)^ is prepared in two dimensions, and the x- and y-axes represent the z^(3)^µ values in the first and second dimensions, respectively. The gray arrow indicates the direction in which z^(3)^ changed over time during data assimilation.

Subsequently, the capacity of the proposed model to estimate underlying latent state changes of observed ECoG signals in real-time was evaluated using ECoG data that transition from an awake state to an anesthetized state (or vice versa) in test individuals. Figure 2a presents an example of test results utilizing ECoG data during the transition from wakefulness to anesthesia. This figure plots observed and predicted ECoG signals (for 5 of 20 channels), their prediction errors, and the time series of latent states across the first to third levels (z^(1)^ to z^(3)^). A comparison between observed and predicted ECoG waveforms reveals successful simulation of ECoGs by the model.

Temporal changes in the latent state, reflecting data assimilation, are rapid at lower levels and slow at higher levels, depending on the time scale. Notably, at the highest level (z^(3)^), a switch in values can be seen in response to the transition from awake to anesthetized states (as enclosed in the box in Fig. 2a), suggesting that the value of z^(3)^ indicates global changes in brain state between wakefulness and anesthesia. Dynamic alterations in these values throughout these processes are depicted in a Supplementary Movie 1.

During data assimilation, changes in latent states z^(3)^ (two-dimensional) were plotted in gray on the scatter plot in Fig. 2b. Red and cyan points represent the values of z^(3)^ for anesthetized and awake conditions, respectively, as obtained through training. These observations revealed that clusters of latent states corresponding to anesthesia and awake conditions appeared to emerge. The trajectory of z^(3)^ across these clusters during data assimilation indicates that the model accurately estimated the transition from the brain states of anesthesia to wakefulness. To quantitatively validate this, data assimilation simulations were conducted using multiple ECoG sequences from test individuals, including transitions from wakefulness to anesthesia and vice versa. Using the k-Nearest Neighbors measure (See Methods for details), we evaluated whether the z^(3)^ states during data assimilation were appropriately positioned in clusters of wakefulness or anesthesia formed during training. These results showed that the proportion of correct cluster localization was, on average, 0.78 (SD 0.14) before the condition switch, and 0.68 (SD 0.11) after the switch (Refer to Supplementary Table 1 for the other clustering metric). These findings suggest that the z^(3)^ states during generation of ECoG data for test individuals transitioning between wakefulness and anesthesia states can estimate these conditions.

### Virtual intervention to z^(3)^ (virtual drug administration)

The preceding analysis revealed that estimating latent states z^(3)^ from a test individual’s ECoG signals allows for prediction of anesthetized and awake conditions. Subsequently, we investigated whether virtual ECoG waveforms, generated by setting this estimated z^(3)^, accurately reflect characteristics of anesthetized and awake ECoGs. This approach, with z^(3)^ as a virtual intervention target, is intended to impact all hierarchical levels and brain regions downstream of z^(3)^, simulating administration of a drug that influences the whole brain. Evaluation of virtual ECoG leveraged a discriminator (3D convolutional neural network^21^) that was designed to differentiate between anesthetized and awake conditions (See Methods for details.). This classifier demonstrated a high level of accuracy, with 99.6% and 98.1% for classification of observed anesthetized and awake ECoG signals. When this discriminator was applied to virtual ECoGs generated by setting the estimated z^(3)^ for anesthetized and awake conditions in test individuals, virtual ECoGs were correctly classified into these respective conditions with high accuracies of 0.89 and 0.96. These results indicate that virtual ECoG signals generated by targeting z^(3)^ in virtual interventions accurately reflect ECoG characteristics of both anesthetized and awake conditions.

### Functional Analysis of z^(2)^ (functional network analysis)

We next explored representations that emerge in latent states of intermediate levels (z^(2)^) following training. The current V-RNN model incorporates multiple latent variables at the second level (z^(2)^), each independently influencing deterministic units in the first level (d^(1)^) corresponding to various brain regions. Hence, each latent variable z^(2)^ is supposed to independently govern a distinct functional network. To analyze how these multiple functional networks change between anesthesia and wakefulness, we calculated transfer entropy as a measure to quantify the strength of control from z^(2)^s to d^(1)^s. Transfer entropy is a measure of the amount of information transfer between two sequences based on information theory. This virtual intervention experiment is designed to simulate interventions targeting functional networks, such as connectivity neurofeedback and DecNef^22,23^.

**Fig. 3:**
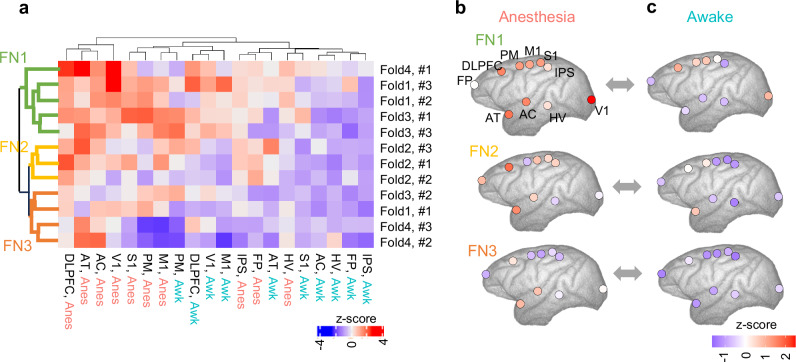
Transfer Entropy Analysis. **a** This heatmap represents transfer entropy values from z^(2)^ latent variables to d^(1)^ in various brain regions. Rows in the heatmap correspond to z^(2)^ latent variables used in calculation of transfer entropy. These rows represent 12 unique z^(2)^ states, derived from four variations in the training dataset during cross-validation (denoted as Fold 1-4), three z^(2)^ latent variables per one model (denoted as #1-3). Columns indicate brain regions associated with d^(1)^ activities utilized in transfer entropy calculations, presented for two conditions: anesthetized (Anes) and awake (Awk). Brain regions are displayed with the following abbreviations: FP, frontal pole; DLPFC, dorsolateral prefrontal cortex; PM, premotor cortex; M1, primary motor cortex; S1, primary somatosensory cortex; IPS, intraparietal sulcus; AT, anterior temporal cortex; AC, auditory cortex; HV, higher visual cortex; and V1, primary visual cortex. The heatmap color scale with transfer entropy values (z-score) from each z^(2)^ to the corresponding brain region d^(1)^, indicating the extent of control (or information flow). Hierarchical clustering was performed on rows and columns of this heatmap matrix, and based on these clustering results, Functional Network (FN) 1, FN2, and FN3 were defined. **b, c** Mapping of transfer entropy values from z^(2)^ to d^(1)^ in different brain regions for FN1, FN2, and FN3.

In the current V-RNN model, d^(1)^s compose 10 brain region modules. Therefore, when provided with two conditions—anesthesia and wakefulness—transfer entropy values from a single z^(2)^ to each brain module (d^(1)^) are obtained as a 20-dimentional vector (10 brain regions×2 conditions). Additionally, the proposed model incorporates three units in z^(2)^, and cross-validation was conducted for four individuals, resulting in separate training of four networks. Consequently, a total of 12 vectors (3 units × 4 networks) were obtained. These vectors were subsequently sorted through hierarchical cluster analysis (Fig. 3a). Focusing on the row direction in Fig. 3a, it appears that transfer entropy values from z^(2)^ to d^(1)^ tend to be higher during the anesthetized state compared to the awake state. Additionally, transfer entropy values from z^(2)^ to d^(1)^ are organized into three distinct clusters, as illustrated by the dendrogram colored in green, yellow, and red in Fig. 3a. These clusters are labeled as functional networks (FN) 1, FN2, and FN3, respectively. These networks plausibly represent networks required to generate ECoG signals under different levels of consciousness during anesthesia and wakefulness. Figure 3b, c visualize the average transfer entropy from z^(2)^ to d^(1)^ in FN1, FN2, and FN3 on brain maps during anesthetized and awake conditions, respectively. This visualization reveals that network control over each brain region differs between anesthetized and awake conditions and that these variations are specific to each FN. Specifically, regions with remarkably different transfer entropy values between anesthetized and awake states include a broad area encompassing primary visual cortex (V1) in FN1, the dorsolateral prefrontal cortex (DLPFC) and parietal lobe in FN2, and the temporal lobe in FN3.

Subsequently, we investigated the impact of these functional networks (FNs) on changes in ECoG patterns across different levels of arousal through a virtual intervention targeting z^(2)^. For this virtual intervention, z^(3)^ states were set to those of an awake condition, under which ECoG signals characteristic of wakefulness would be generated in the absence of intervention. Thereafter, a virtual intervention was conducted on z^(2)^ states, altering each unit individually to a state representative of anesthesia. By applying ECoG signals generated through this intervention to the discriminator described in the previous section, we can assess the extent to which generated ECoG signals transitioned toward those characteristic of anesthesia. This approach enables evaluation of causal effects of individual FNs on generation of anesthetic ECoG signals. The proportion of anesthetic ECoG signals generated was 0.25 ( ± 0.20) for FN1, 0.22 ( ± 0.20) for FN2, and 0.49 ( ± 0.35) for FN3. Conversely, a similar procedure was conducted by setting the state of z^(3)^ to an anesthetized state and intervening on z^(2)^ to alter it to an awake state. As a result, the proportion of wakeful ECoG signals generated was 0.75 ( ± 0.18) for FN1, 0.58 ( ± 0.18) for FN2, and 0.56 ( ± 0.17) for FN3. These findings indicate a tendency for a higher proportion of wakeful ECoG signals to be generated when z^(2)^ is set to an awake state, compared to the proportion of anesthetic ECoG signals generated when z^(2)^ is set to an anesthetized state. Moreover, the proportion of ECoG signals that were altered to either anesthetized or wakeful states following intervention appeared to vary among FNs.

### Functional Analysis of z^(1)^

As in the scheme outlined in the previous section, we next examined the impact of latent states at a regional level (z^(1)^) on changes in ECoG pattern across different arousal levels in a virtual intervention experiment. Initially, by setting z^(3)^ for the awake condition and z^(1)^ for the anesthetized condition in a target brain region, we generated ECoG signals and used the discriminator for condition differentiation. Similarly, we performed another generation where z^(3)^ was set to the anesthetized condition and z^(1)^ to the awake condition. These virtual interventions at z^(1)^ are designed to simulate treatments targeting specific brain regions, including methods such as tDCS and TMS. Table 1 presents the proportion of generated ECoG signals that exhibited an intervention effect in specific brain regions following the virtual intervention. The proportion of ECoG signals classified as anesthetized following the intervention of setting z^(1)^ for the anesthetized condition ranged from 0.24 to 0.38, whereas the proportion of awake ECoG signals generated after setting z^(1)^ for the awake condition was relatively higher, ranging from 0.43 to 0.52. To examine whether there are differences in intervention effects across brain regions (z^(1)^), we constructed a two-way ANOVA model with the proportion of anesthesia and wakefulness ECoG as the dependent variable, and brain regions and individuals as independent variables. These results showed no statistically significant differences in intervention effects across brain regions, whether the intervention was designed to induce anesthesia or wakefulness (main effect of brain region: P = 0.122 and P = 0.644, respectively).

**Table 1:** Proportion of generated ECoGs with intervention effects by intervention brain region

Brain region		FP	DLPFC	PM	M1	AT	AC	HV	S1	IPS	V1
Set z^(1)^ latent states: anesthesia^a^	Mean	0.27	0.27	0.27	0.24	0.26	0.29	0.38	0.28	0.36	0.24
	SD	0.21	0.16	0.16	0.17	0.18	0.22	0.33	0.17	0.24	0.18
Set z^(1)^ latent states: wakefulness^b^	Mean	0.43	0.48	0.43	0.50	0.46	0.41	0.52	0.48	0.44	0.44
	SD	0.09	0.11	0.10	0.04	0.13	0.11	0.18	0.04	0.18	0.09

^a^ The proportion of generated ECoG signals classified as being in an anesthetized state by the discriminator when the z^(1)^ of each brain region is individually set to an anesthetized state, while z^(3)^ is set to an awake state.

^b^ The proportion of generated ECoG signals classified as being in an awake state by the discriminator when the z^(1)^ of each brain region is individually set to an awake state, while z^(3)^ is set to an anesthetized state.

SD standard deviation, FP frontal pole, DLPFC dorsolateral prefrontal cortex, PM premortor cortex, M1 primary motor cortex, S1 primary somatosensory cortex, IPS intraparietal sulcus, AT anterior temporal cortex, AC auditory cortex, HV higher visual cortex, V1 primary visual cortex.

### Individuality analysis

**Fig. 4:**
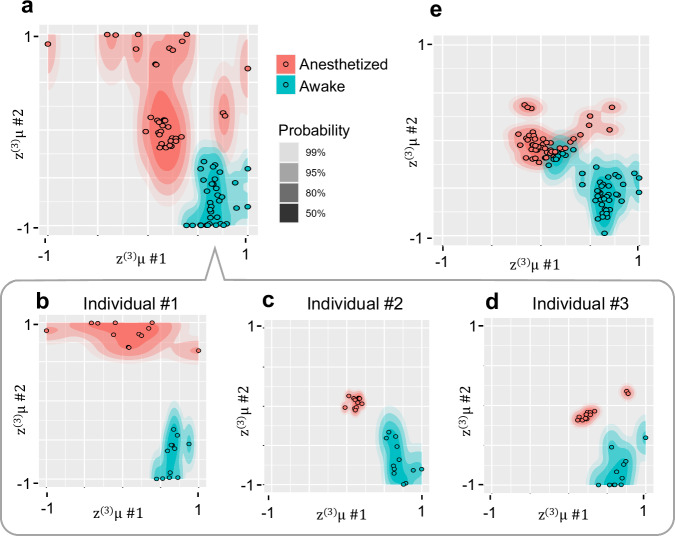
Individual Distribution of Latent states. **a** Distribution of z^(3)^μ post-training, as shown in Fig. 2b, overlaid with an estimated probability density plot. Each layer of varying shades represents boundaries of corresponding probability levels. Estimation of bivariate probability density utilized the kernel density estimator from the “ggdensity” package in R. **b–d** Distributions of z^(3)^μ depicted in Fig. 3a are shown separately for each of three individuals in the training data. **e** Shows the distribution of z^(3)^μ estimations from data assimilation applied to ECoG sequences from a test individual.

Finally, we assessed whether the proposed model could reflect individual characteristics, focusing on z^(3)^ representation. Figure 4a presents an example of the estimated probability density distribution of z^(3)^ states for awake and anesthetized conditions of the three individuals used in training. Figure 4b, c and d identify which individual corresponds to each point among these training results, whereas Fig. 4e estimates the z^(3)^ states of a test individual. The distribution of z^(3)^ under anesthesia appears to vary among individuals, whereas the distribution under awake conditions appears to overlap. Quantitative evaluation based on cross-validation confirms individual-specific clustering under anesthesia (average silhouette width 0.285 > 0, P < 2.2×10^-16^), whereas no statistically significant cluster structure was observed under wakefulness (−0.10, P > 0.05). Thus, in terms of z^(3)^ representation, individual differences are self-organized under anesthesia, whereas individual representations are not distinctly observed under wakefulness. Subsequently, we examined whether waveforms of virtual ECoGs generated based on the estimated z^(3)^ reflect individual characteristics. Evaluation of virtual ECoG signals leveraged a discriminator (3D convolutional neural network^21^) that was designed to distinguish among the four individuals used in the experiment (details in Methods). These results indicate that the proportion of correctly identified ECoG signals generated for test individuals was 0.13 under anesthesia and 0.07 when awake, suggesting that ECoG characteristics of the test individuals were not sufficiently captured.

## Discussion

In this study, we developed a digital twin brain prototype capable of generating ECoG signals from test individuals with high precision in real-time. This was achieved by employing a V-RNN with latent variables possessing temporal and spatial hierarchical structures, which updates these latent variables sequentially through data assimilation from observed ECoGs. Examination of the latent variable representation that facilitated such precise simulations revealed its self-organized structure at different hierarchical levels. Specifically, at higher levels of latent states (z^(3)^), clusters reflective of global brain states such as anesthesia and wakefulness, as well as individual uniqueness, were observed. Clusters of these z^(3)^ latent states enabled appropriate updates of latent states during data assimilation of ECoG signals from test individuals, leading to successful virtual drug administration simulations. Transfer entropy analysis between intermediate (z^(2)^) and lower (d^(1)^) hierarchical levels indicated emergence of activity patterns that control local brain regions corresponding to functional networks. Virtual interventions on these functional networks demonstrated that the effect of altering generated ECoGs from wakefulness to anesthesia varied significantly across functional networks.

There have been previous efforts to achieve real-time simulation of ECoG and EEG signals that can be compared to the current simulator. For example, in pursuit of EEG signal simulation, dynamics of average postsynaptic potentials (PSPs) in excitatory and inhibitory interneuron populations under electrodes have been modeled using differential equations^10,24,25^. These equations incorporate synaptic gain and time constants as time-varying parameters, with these parameters being inferred in real-time from observed EEG signals. Ultimately, the sum of the PSPs of excitatory and inhibitory interneurons is outputted as the prediction of the EEG signal. While this modeling approach is notably advantageous for its low computational cost, it is primarily designed for simulating a single-channel EEG signals^24^, posing limitations on capturing intricate interactions among various brain regions. Concomitant with such real-time-focused model development, there have been increased attempts to create models that generate interactions among brain regions, reflecting individuality based on brain imaging^9,11,26,27^. These efforts often employ graph network models, representing interactions among brain regions with differential equations that primarily involve linear transformations of state variables of brain regions. Parameters for these linear transformations, such as weights or distances of connections between brain regions, are determined based on diffusion brain MRI and related methods, which measure brain structures, allowing model individualization^26^. These models are frequently used to simulate the BOLD signals in fMRI. A notable feature of these models is that model structure can be determined based on the actual brain structure of the individual, allowing discussion of the relationship between brain structure and brain function^9,26^. On the other hand, technical challenges remain in realizing real-time data assimilation. A shared attribute among models that are currently extensively utilized is explicit a priori formulation of dynamics specific to each brain signal type using differential equations^24,26^. In contrast, the primary characteristic of our model using artificial neural networks is their data-driven training, offering substantial flexibility in handling input signals and facilitating modeling of diverse modalities, including simultaneous modeling of multiple signal types. In scenarios involving processing of brain signals, such as EEG, MEG, and fMRI, coupled with sensory input signals (visual and auditory), the proposed model’s general applicability could be valuable. On the other hand, reduction of computational cost in the real-time PSP simulator and incorporation of individual brain structures into the architecture of the graph network model are notable features of these previous models, suggesting that combining insights from these models can lead to refinement of the proposed model.

In this section, we compare our findings with related studies that have utilized the same ECoG dataset (Neurotycho dataset^16^). Vetter et al. employed a conditional denoising diffusion probabilistic model to generate ECoG signals^28^. Although their work did not focus on real-time generation, they successfully generated ECoG signals over a prolonged period of 1.5 h during the transition from wakefulness to anesthesia. This approach demonstrates a higher level of scalability compared to our analysis. Their method achieved 83% accuracy in distinguishing between anesthesia and wakefulness using log-likelihood, which is comparable to results obtained in our study. Furthermore, power spectrum density plots illustrated from their generated ECoG signals exhibited similar distributions to observed ECoG signals, consistent with results in our study (Supplementary Fig. 3). In another related study, Chen et al. applied a graph convolutional network to classify observed ECoG signals into three classes: awake, moderate sedation, and deep sedation, achieving an accuracy of 92.75%. Our approach, utilizing a three-dimensional convolutional neural network (CNN) as a discriminator, achieved comparable accuracy of 98.1-99.6% in the binary classification between wakefulness and anesthesia. These findings indicate that the generative performance of PV-RNN using data assimilation, as well as the discriminative performance using three-dimensional CNN are on par with those of previous studies.

The proposed model not only enables real-time, high-precision simulation of brain signals, but also has potential to offer new insights into mechanisms underlying changes in brain function during anesthesia, through functional analysis. In this study, we observed that Transfer Entropy from z^(2)^ to various brain regions tends to be higher under anesthesia compared to the awake state. Additionally, we found that the proportion of ECoG signals generated for each FN differed between anesthetized and awake states. The biological manner in which whole-brain networks change and lead to alterations in consciousness following administration of anesthetics remains largely unknown in the field of neuroscience^29,30^. Therefore, the hypothesis presented in this study regarding changes in information processing between anesthesia and wakefulness should be further investigated in the future. However, we believe that our current findings do not contradict existing, albeit fragmented, results from biological studies in neuroscience. For example, increased connectivity from subcortical regions, such as the thalamus to the cerebral cortex has been reported^31^, which may be related to our observation of increased Transfer Entropy from z^(2)^ to d^(1)^. Furthermore, regions such as the DLPFC, temporal cortex, and V1, which showed significant changes in Transfer Entropy values between anesthesia and wakefulness in our FN analysis, have been identified as regions affected by anesthesia in functional studies^29^. We anticipate that combining our virtual intervention experiments with biological research in a bidirectional manner, in which hypotheses and validations inform each other, will contribute to a deeper understanding of mechanisms by which anesthesia alters levels of consciousness.

Furthermore, results of the functional analysis showed that virtual interventions on z^(2)^ led to relatively large differences in generation of anesthesia and awake ECoG signals among FNs, whereas interventions on z^(1)^ resulted in smaller differences among brain regions. A key point in interpreting these results is that z^(2)^ controls integration of multiple regions, whereas z^(1)^ only controls individual regions. It is important to note that information from z^(2)^ through d^(2)^ and information from z^(1)^ are fed separately into d^(1)^ (Fig. 1a). z^(2)^ is designed to represent integrated components of information that span multiple regions, while z^(1)^ is designed to represent components of information that are independent of other regions. The current findings suggest that information critical for transforming generated ECoG signals under anesthesia or awake is likely represented as integrated information across multiple regions. This interpretation is consistent with previous neuroscience research, demonstrating that neurophysiological markers across a wide range of brain regions are altered during administration of anesthetics^29^.

In this study, generated ECoG signals were classified with high accuracy by the discriminator as either anesthetized or awake conditions. To explore underlying reasons for this success, we examined the power spectrum density of both observed and generated ECoG signals across different brain regions (Supplementary Fig. 2). We found that the power spectrum of observed ECoG signals was generally higher during anesthesia compared to wakefulness in most brain regions. However, in the higher visual cortex (HV) and primary visual cortex (V1) regions, differences in the power spectrum between anesthesia and wakefulness were relatively small. Generated (predicted) ECoG signals successfully replicated these regional differences in the power spectrum between anesthesia and wakefulness, which may explain the high accuracy of the discriminator in classifying generated ECoG signals. On the other hand, generated ECoG signals tended to have slightly lower power spectra compared to observed ECoG signals, suggesting that there is potential to further improve performance by updating network structure parameters and training data (also, see the Limitations section of the Discussion).

In the data assimilation process of the current experiment, predictions were only made one time step ahead of the time window. However, by extending the range of prior generation (Eqs. (1–4) in the Methods), it is possible to predict further into future time steps. We investigated the relationship between the number of future time steps predicted and the corresponding prediction error (see Supplementary Table 2). The results showed that the rate of increase in prediction error was gradual as the prediction horizon extended from one step to 100 steps ahead. This suggests that the model used in this study may also be applicable for predicting multiple time steps into the future.

We examined the impact of the number of updates to latent states and the width of the time window on the performance of ECoG signal generation through data assimilation (Supplementary Tables 3-6). Performance metrics for ECoG signal generation included the prediction error and accuracy of inferring z^(3)^. Accuracy of z^(3)^ inference was assessed, as in the Results, by evaluating the proportion of inferred z^(3)^ during data assimilation apportioned to the correct clusters (anesthetized and awake clusters obtained from training). These results indicated that reducing update times from the original 100 to 50, 25, and 10 led to an increase in prediction error and a decrease in the proportion of z^(3)^ in the correct cluster. In contrast, reducing the time window width from the original 500 to 100 did not significantly alter the prediction error or the proportion of z^(3)^ in the correct cluster. However, further reducing the time window below 50 resulted in a deterioration of these metrics. These findings suggest that if computational speed is a priority, reducing the time window might be a more effective strategy for minimizing performance degradation compared to reducing the update times.

In this study, we conducted a virtual ECoG generation experiment on test individuals to examine whether individual characteristics were replicated. These results confirmed the emergence of subject-specific clusters in the higher-order latent state z^(3)^. However, when applying a discriminator to generated ECoG signals, the proportion that correctly identified the corresponding individual was low. Specifically, the proportion of signals classified as originating from the test subjects was below the chance level, with many signals classified as belonging to the training subjects. This suggests overfitting. To further explore this, we generated power spectrum density plots for both generated and observed ECoG signals (Supplementary Fig. 3). These plots suggest that differences in power spectra among individuals in observed ECoG signals are smaller than differences between wakefulness and anesthesia. While the predicted ECoG successfully reproduced the power spectrum differences between wakefulness and anesthesia, it did not adequately capture inter-individual differences in the power spectrum. For instance, the generated ECoG for an individual with relatively high observed ECoG power (Test individual #4) only produced a power distribution similar to other individuals. Additionally, there seems to be a general tendency for generated ECoG signals to exhibit lower power compared to observed ECoGs across all subjects. These indicate that while the current generated ECoG successfully reproduces the relationship between power spectra of wakefulness and anesthesia, it fails to adequately replicate inter-individual power spectrum relationships. Several factors may account for this outcome. First, generating ECoG signals for test individuals using only training data could be inherently difficult, as training data lack enough detail to accurately capture unique characteristics of test individuals. Introducing techniques such as fine-tuning, which adjusts the model using a subset of test subject data, could improve accuracy. Second, the network architecture used in this study may not have been suitable for reproducing individual-specific characteristics. The current model does not include latent units explicitly designed to represent individuality. While individual differences appeared to be encoded in the global latent state, this state also represented information related to wakefulness and anesthesia, leading to mixed representations. Incorporating a network architecture with latent units specifically dedicated to individual differences could enable generation of ECoG signals that better capture individuality. In addition to improving network structures, future studies are expected to explore how individuality is represented when increasing the number of training individuals or when dealing with human brain signals, instead of those of macaques.

It is important to note that the current model is not identifiable, meaning that there is no guarantee that latent variables can be uniquely inferred from observed signals. This issue of identifiability may be related to the slight overlap observed in latent variables inferred through data assimilation across conditions (anesthesia and wakefulness) and among individuals, as well as insufficient distinctiveness of individual characteristics in generated ECoG signals. To achieve identifiability, models incorporating constraints on prior distributions or generative functions have been proposed^32,33^. Incorporating such constraints to achieve identifiability in our model remains a critical challenge for future research.

In the proposed model, the process of multiple updates to latent states during data assimilation incurs significant computational costs. One promising approach to reduce these costs is amortized inference, a method in which current latent states are directly predicted from observed data through forward computation^34,35^. This technique involves pre-training a parameterized function (such as a neural network or encoder) that directly maps observed data to current latent states, thereby enabling inference of these states without the need for multiple update iterations. While this method can effectively reduce computational costs, it may introduce generalization issues, commonly known as the “amortization gap,” due to the fact that the encoder, which infers latent states from observed data, is not updated during testing. Recent advances have focused on integrating amortized inference with iterative inference to achieve both rapid computation and improved generalization performance^36–38^. In the future, it may be possible to apply this integrated approach to our model, enhancing its efficiency and generalization capabilities.

The proposed model can be discussed in relation to modeling of cognitive processes from the perspective of brain computation theory. The model employs free energy minimization through parameter optimization, which is a core aspect of the free energy principle derived from Bayesian brain theory^39,40^. This theory hypothesizes that the brain’s information processing is analogous to data assimilation. Indeed, similar RNN models have been utilized in computational modeling and neurodevelopmental robotics research to understand cortical information processing, cognitive functions, and psychiatric symptoms^41–45^. The present research may be interpreted as demonstrating that the free energy principle-based approach is beneficial for simulating not only cognitive processes, but also brain signals. As future challenges, it is anticipated that the proposed model will first be extended to other brain signals, such as EEG and fMRI, which can be measured non-invasively, but which contain considerable noise. Furthermore, integrating modalities such as sensory input and motor output alongside brain signals is expected to enable development of models capable of comprehensively simulating cognitive processes. This approach indicates the potential to uncover latent state changes underlying cognitive features in neuropsychiatric disorders and to facilitate virtual intervention experiments. Furthermore, by advancing virtual intervention experiments targeting z^(1)^ (local regions), it becomes possible to simulate both virtual interventions in specific brain regions and resulting functional changes across the whole-brain network. For example, perturbation simulation^46^ can be employed to examine the impact of impairments in specific brain regions on overall functionality of the brain network. Additionally, this approach is expected to be applicable to simulations of neuro-modulation treatments^12,47^ conducted for mental and neurological disorders.

## Methods

### Data and preprocessing

In this study, we utilized electrocorticogram (ECoG) data obtained from the publicly available Neurotycho database^15,16,48,49^, specifically focusing on recordings from macaque monkeys. These shared data comprise recordings from 128 electrodes distributed across the entire hemisphere. Due to computational resource constraints, we selected 20 electrodes for further analysis. The selection process sought to cover the entire brain by including electrodes from all major lobes—frontal, temporal, parietal, and occipital. Additionally, to ensure that various brain functions were represented, we chose regions associated with higher-order cognitive functions, associative processing, sensory perception, and motor functions. Consequently, we selected two channels from each of the following ten representative brain regions, resulting in a total of 20 channels: frontal pole (FP), primary visual cortex (V1), higher visual cortex (HV), auditory cortex (AC), primary somatosensory cortex (S1), primary motor cortex (M1), intraparietal sulcus (IPS), premotor cortex (PM), dorsolateral prefrontal cortex (DLPFC), and anterior temporal cortex (AT).

Preprocessing of ECoG data encompassed re-referencing, outlier exclusion, and linear normalization, which were performed on an individual basis. During the re-referencing process, the Common Median Reference method was applied. Outlier exclusion adhered to protocols from the preceding study using the same dataset^16^, in which data were divided into 2000 time-step (2-second) bins, and bins with values exceeding eight standard deviations, as well as their immediate neighbors, were excluded. Consequently, 1.4% of data under anesthesia and 8.6% in awake macaques were excluded. Linear normalization was performed to shift the original range of [-1000μV, 1000μV] to [-0.8, 0.8]. This normalization process ensured that ECoG signal input fell within the range of [-1, 1], which is the range that our model can handle. This range [-1000 μV, 1000 μV] was selected based on the observation that over 99% of ECoG data from separate macaque experiments, conducted under different anesthesia conditions (single-agent ketamine), fell within this range^15^. This suggests that the selected range is a reasonable estimate for typical ECoG values in macaques. Additionally, ECoG data were inputted into the model at the original sampling rate of 1000 Hz, without downsampling. This decision was based on preliminary investigations, which indicated that downsampling could result in the absence of distinct clusters corresponding to awake and anesthetized states in the latent variable.

Following these preprocessing steps, data were segmented again into 2000 time-step (2-second) intervals from which twelve anesthetized and twelve awake sequences were randomly selected from each of the three training individuals, totaling 72 sequences (144,000 time steps,144 s) used for training. The rationale for choosing this sequence length is based on preliminary analysis, which revealed that clustering of z^(3)^µ associated with anesthesia and wakefulness was weak when the sequence length was less than 2000 time steps. Then, to validate our model’s efficacy on test individual ECoG data, we devised three sets of test data for different experiments. For the real-time latent state estimation experiment, we crafted 25 sequences by concatenating ECoG signals: the first 2500 time steps were drawn from randomly selected awake sequences, whereas the latter 1500 time steps were derived from randomly chosen anesthetized sequences. Another 25 sequences were similarly prepared, but with the order of states reversed, totaling 50 sequences. This disparity in time steps between the first and second halves accounts for consideration of a time window width $H$ of 500 time steps. For sequence concatenation, we employed the linear crossfade method, a tapering technique frequently used in similar studies^50,51^. The tapering width was set to 30 ms to minimize the effects of tapering, including on high-frequency components present in ECoG signals. For the virtual intervention experiment to z^(3)^ and individuality analysis, we randomly selected 100 sequences of ECoG signals truncated at 2000 time steps, 50 under anesthesia and 50 while awake. Similarly, for virtual intervention experiments on z^(2)^ or z^(1)^, we randomly selected 50 sequences of ECoG signals truncated at 2000 time steps, 25 under anesthesia and 25 while awake.

### System Overview

This simulator’s main component employed a V-RNN, characterized by its spatial and temporal hierarchy. This hierarchy consists of the following three levels: a local region level (the first level), corresponding to modules for each of the 10 brain regions, operating on rapid changes at short time scales; the functional network level (the second level), consisting of a single module that controls local region modules and operates on slower changes over longer time scales; and the global state level (the third level), also a single module, which maintains constant latent states during generation of sequence predictions to map to sequential patterns of the target ECoG at an abstract level. These hierarchical structures are facilitated by each module comprising probabilistic latent units $z$ and deterministic units $d$ with time-scale-specific dynamics. In this model, generation of ECoG signals is characterized by top-down prediction, where information flows from latent units $z$ to deterministic units $d$, descending to lower levels. Dynamics in these top-down predictions are acquired through a data-driven training phase. Following the training phase, a data assimilation phase utilizing ECoG data from test individuals facilitates real-time simulations. In the data assimilation process, initially, the latent states ${z}_{t}$ at each hierarchical level for time step $t$ are estimated as “priors.” Leveraging these priors, the model conducts top-down prediction of ECoG signals ${\hat{x}}_{t}$. Subsequently, the observed ECoG signal ${x}_{t}$ is inputted. Based on the prediction error (the difference between ${\hat{x}}_{t}$ and ${x}_{t}$), latent states ${z}_{t}$ are updated, enabling a bottom-up estimation of the “posterior.” This posterior informs the next latent state ${z}_{t+1}$ estimation, which becomes the new prior. This iterative process of top-down prediction and bottom-up posterior updates enables real-time estimation of latent states and the generation of virtual ECoG signals.

### Top-down prediction generation

In top-down prediction, the prior and posterior of latent states $z$ are utilized to derive deterministic states *d*, enabling ECoG signal prediction $\hat{x}$. To illustrate this process, we initially discuss calculation of the prior for latent states $z$. Specifically, we consider the prior distribution $p\left({z}_{t,i}^{\left(s\right)}\right)$ for the *i* th latent state ${z}_{t,i}^{\left(s\right)}$ at time step *t* of the *s* th target sequence, at local region and functional network levels. This prior distribution is assumed to follow a Gaussian distribution, where the mean ${\mu }_{t,i}^{\left(s\right),p}$ and standard deviation ${\sigma }_{t,i}^{\left(s\right),p}$ are derived from the previous deterministic states ${d}_{t-1,j}^{\left(s\right)}$.

1 $$p\left({z}_{t,i}^{\left(s\right)}\right)=p\left({z}_{t,i}^{\left(s\right)}|{d}_{t-1,j}^{\left(s\right)}\right){{=}}{\mathcal{N}}{{(}}{z}_{t,i}^{\left(s\right)};{\mu }_{t,i}^{\left(s\right),p},{\sigma }_{t,i}^{\left(s\right),p})$$

2 $${\mu }_{t,i}^{\left(s\right),p}={tanh} \left(\sum _{j}{w}_{{ij}}{d}_{t-1,j}^{\left(s\right)}\right)$$

3 $${\sigma }_{t,i}^{\left(s\right),p}={exp} \left(\sum _{j}{w}_{{ij}}{d}_{t-1,j}^{\left(s\right)}\right)$$

4 $${z}_{t,i}^{\left(s\right)}={\mu }_{t,i}^{\left(s\right),p}+{\sigma }_{t,i}^{\left(s\right),p}\times \varepsilon$$

Here, $j$ is an index representing deterministic units in the same module as ${z}_{t,i}^{\left(s\right)}$. In Eq. (4), $\varepsilon$ is sampled from the ${\mathcal{N}}(\mathrm{1,0})$ distribution to obtain the latent state ${z}_{t,i}^{\left(s\right)}$. As described above, the latent state ${z}_{t,i}^{\left(s\right)}$ changes at each time step at local region and functional network levels, but at the global state level, the ${z}_{t,i}^{\left(s\right)}$ is kept constant during prediction generation. For all levels, the prior distribution for the initial time step of latent states is set as ${\mathcal{N}}({0,1})$. Use of the Gaussian distribution is based on the central limit theorem^52,53^.

Next, the posterior distribution $q\left({z}_{t,i}^{\left(s\right)}\right)$ of latent states is calculated as the conditional probability dependent on prediction error signals ${e}_{t:{T}^{(s)}}^{\left(s\right)}$.

5 $$q\left(z_{t,i}^{(s)} \mid e_{t:T^{(s)}}^{(s)}\right) = \left\{\begin{array}{ll}q\left(z_{t-1,i}^{(s)} \mid e_{t-1:T^{(s)}}^{(s)}\right) = q\left(z_{1,i}^{(s)} \mid e_{1:T^{(s)}}^{(s)}\right) = {\mathcal{N}}\left(z_{1,i}^{(s)}; \mu_{1,i}^{(s),q}, \sigma_{1,i}^{(s),q}\right), & \text{if}\enspace i \in I_{Gz}, \\{\mathcal{N}}\left(z_{t,i}^{(s)}; \mu_{t,i}^{(s),q}, \sigma_{t,i}^{(s),q}\right), & \text{if}\enspace i \in I_{Fz}, I_{L(n)z}.\end{array}\right.$$

6 $${\mu }_{t,i}^{\left(s\right),q}=\tanh \left({a}_{t,i}^{\left(s\right),\mu }\right)$$

7 $${\sigma }_{t,i}^{\left(s\right),q}={exp} \left({a}_{t,i}^{\left(s\right),\sigma }\right)$$

8 $${z}_{t,i}^{\left(s\right)}={\mu }_{t,i}^{\left(s\right),q}+{\sigma }_{t,i}^{\left(s\right),q}\times \varepsilon$$

Here, ${T}^{\left(s\right)}$ represents the length of the $s$ th target ECoG sequence. ${a}_{t,i}^{\left(s\right)}$ is defined as the unit’s adaptive internal state that represents the posterior distribution. ${a}_{t,i}^{\left(s\right)}$ is dynamically updated at each timestep in every target ECoG sequence throughout the processes of learning and data assimilation. This updating process is influenced by the prediction error signal ${e}_{t:T}$, which is propagated via a back-propagation-through-time (BPTT) algorithm. Thus, as inferred from Eqs. (6) and (7), the posterior distribution of latent states may be conceptualized as a prediction-error-related unit state.

Based on calculated latent states ${z}_{t,i}^{\left(s\right)}$ as described above, internal states ${h}_{t,i}^{\left(s\right)}$ and outputs ${d}_{t,j}^{\left(s\right)}$ of deterministic units are computed as follows. This process involves deriving ${h}_{t,i}^{\left(s\right)}$ from the previous deterministic output ${d}_{t-1,j}^{\left(s\right)}$ and latent states ${z}_{t,j}^{\left(s\right)}$ in the same module, alongside inputs from a higher-level module (Supplementary Fig. 5).

9 $${h}_{t,i}^{\left(s\right)}=\left\{\begin{array}{ll}\frac{1}{{\tau }_{i}}\left(\mathop{\sum}\limits_{j\in {I}_{{Fd}}}{w}_{{ij}}{d}_{t-1,j}^{\left(s\right)}+\mathop{\sum}\limits_{j\in {I}_{{Fz}}}{w}_{{ij}}{z}_{t,j}^{\left(s\right)}+\mathop{\sum}\limits_{j\in {Gz}}{w}_{{ij}}{z}_{t,j}^{\left(s\right)}+{b}_{i}\right)+\left(1-\frac{1}{\tau }\right){h}_{t-1,i}^{\left(s\right)}, & {\mathrm{if}} \, i\in {I}_{{Fd}},\\ \frac{1}{{\tau }_{i}}\left(\mathop{\sum}\limits_{j\in {I}_{L\left(n\right)d}}{w}_{{ij}}{d}_{t-1,j}^{\left(s\right)}+\mathop{\sum}\limits_{j\in {I}_{L\left(n\right)z}}{w}_{{ij}}{z}_{t,j}^{\left(s\right)}+\mathop{\sum}\limits_{j\in {Fd}}{w}_{{ij}}{d}_{t,j}^{\left(s\right)}+{b}_{i}\right)+\left(1-\frac{1}{\tau }\right){h}_{t-1,i}^{\left(s\right)}, & {\rm{if}} \,i\in {I}_{L\left(n\right)d}. \end{array}\right.$$

10 $${d}_{t,j}^{\left(s\right)}={tanh} \left({h}_{t,i}^{\left(s\right)}\right), {\text{if}} i\in {I}_{{Fd}},{I}_{L\left(n\right)d}.$$

Here, ${{I}_{L\left(n\right)d},I}_{{Fd}},{I}_{{Gd}}$ represent index sets for deterministic units at the local region level, functional network level, and global state level, respectively, while ${{I}_{L\left(n\right)z},I}_{{Fz}},{I}_{{Gz}}$ represent index sets for latent units at their corresponding levels. The $L(n)$ takes on $n=\mathrm{1,2},\ldots ,10$, corresponding to modules representing ten local brain regions. The ${w}_{{ij}}$ refers to the weight of synaptic connections between units. Time constant τ is a predefined hyperparameter, where deterministic units with smaller ${\tau }_{i}$ tend to exhibit rapid state changes, while those with larger ${\tau }_{i}$ tend to show slower state changes^20^. By setting ${\tau }_{i}=2$ at the local region level and ${\tau }_{i}=4$ at functional network level, the model represents changes on a shorter timescale at the lower level and on a longer timescale at the higher level, effectively constructing a temporal hierarchical structure. We selected these ${\tau }_{i}$ values based on pilot experiments that identified τ values yielding the smallest, most stable prediction error. Increasing τ beyond these settings tended to impair prediction of high-frequency signals significantly.

Lastly, prediction ${\hat{x}}_{t,i}^{\left(s\right)}$ of ECoG signals for 20 channels corresponding to 10 brain regions is generated by the respective local brain region module.

11 $${\hat{x}}_{t,i}^{\left(s\right)}=\tanh \left(\sum _{j\in {I}_{L\left(n\right)d}}{w}_{{ij}}{d}_{t,j}^{\left(s\right)}\right), {\text{if}}{i}\in {I}_{L\left(n\right)o}.$$

Here, the ${I}_{L\left(n\right)o}$ denotes index sets of output units in the brain region $L(n)$.

### Parameter updates through bottom-up optimization

Optimization processes in both training and data assimilation phases have been implemented by employing the negative Evidence Lower Bound (ELBO) as the loss function. This negative ELBO is equivalent to the variational free energy^54^ in the context of brain computation theory, and is hereafter identified as ${F}_{t}$. In the framework of V-RNN, ${F}_{t}$ is derived as follows^19^.

12 $${F}_{t}=\frac{1}{2}{\left({x}_{t}-{\hat{x}}_{t}\right)}^{2}+\mathop{\sum }\limits_{l=1}^{3}{W}^{(l)}{D}_{{KL}}\left[{\rm{q}}\left({z}_{t}^{(l)}|{e}_{t:T}\right)\left\|p\left({z}_{t}^{(l)}|{d}_{t-1}^{(l)}\right)\right]\right.$$

The first term of this equation represents the prediction error, while the second term denotes the influence of the prior $p\left({z}_{t}^{(l)}|{d}_{t-1}^{(l)}\right)$ on the update of the posterior ${\rm{q}}\left({z}_{t}^{(l)}|{e}_{t:T}\right)$ of the latent state. Consequently, in an optimization process aimed at minimizing ${F}_{t}$, the update of the posterior progresses in a manner that minimizes the prediction error under the influence of the prior. The hyperparameter ${W}^{(l)}$, referred to as the meta-prior^18^, serves to balance the prediction error and the divergence between the prior and posterior, and can be individually set for each hierarchical level $l$. In this study, we set the meta-prior to a uniform value across all levels (${W}^{(1)}={W}^{(2)}{=W}^{(3)}=0.001$). According to our previous research^18^, if the meta-prior is too small, overfitting occurs, whereas if the meta-prior is too large, the reconstruction error does not decrease sufficiently. This optimization by minimizing ${F}_{t}$ was carried out during both the training and data assimilation phases.

During the training phase, weights $w$ and the posterior are updated to minimize the free energy ${F}_{t}^{(s)}$ over all time steps and target sequences.

13 $$F=\sum _{s\in {I}_{s}}\mathop{\sum }\limits_{t=1}^{{T}^{(s)}}{F}_{t}^{(s)}$$

On the other hand, during the data assimilation phase, only the posterior is updated, while the weights $w$ remain fixed. In this phase, the free energy is summed over the time window spanning the current time step $t$ backward through $H$ time steps.

14 $$F=\mathop{\sum }\limits_{{t}^{{\prime} }=t-H+1}^{t}{F}_{t{\prime} }$$

Utilizing the summed free-energy, the posterior of ${z}_{t-H+1:t}$ in time windows of all modules is updated, with this window advancing over time.

Weights $w$ and posterior latent states $z$, optimized during training and data assimilation, are updated through iterative cycles of top-down prediction generation and bottom-up optimization (BPTT algorithm). $w$ and $z$ (specifically, $\mu$ and $\sigma$) at the $n$-th iteration can be represented as follows:

15 $$w\left(n\right)=w\left(n-1\right)+\Delta w\left(n\right)$$

16 $$z\left(n\right)=z\left(n-1\right)+\Delta z\left(n\right)$$

Here, $\Delta w\left(n\right)$ and $\Delta z\left(n\right)$ are computed using the optimization algorithm (Rectified Adam^55^) based on the gradients of free energy ($\frac{\partial F}{\partial w}$ and $\frac{\partial F}{\partial z}$) .

### Data assimilation

Following the training phase, the data assimilation process is applied to ECoG signals of test individuals. Data assimilation, originally utilized in the field of Earth sciences^56^, has increasingly gained prominence in recent years in the domain of neuroscience, particularly in neural circuit modeling^25,57,58^. It refers to the process of integrating model predictions with real-time observations to estimate latent states. In the context of this study, data assimilation involves an iterative process that includes top-down predictions and bottom-up posterior updates derived from prediction errors. This process occurs in a sliding time window that spans the current time step $$t$$ backward through $$H$$ time steps, and incrementally moves forward as time progresses (Supplementary Fig. 6). The process unfolds as follows: (1) The prior of $${z}_{t}$$ is estimated based on the posterior of the previous time step. (2) Based on this prior of $${z}_{t}$$ and the posterior at earlier time steps ($${z}_{z-H+1:t-1})$$, ECoG signals $${\hat{x}}_{t-H+1:t}$$ in the time window are predicted in a top-down manner. (3) Subsequently, newly observed ECoG signals $${x}_{t}$$ are inputted. (4) At each time step in the time window, the free-energy $$F$$ is calculated based on the prediction error (the discrepancy between $$x$$ and $$\hat{x}$$). (5) To minimize the free-energy $$F$$, latent states $${z}_{t-H+1:t}$$ are updated as the posterior. (6) Based on this updated posterior, top-down predictions for $${\hat{x}}_{t-H+1:t}$$ are performed again. This process of bottom-up posterior updates (4)(5) and top-down prediction (6) is repeated a predetermined number of times. Subsequently, the process returns to (1), where, from the posterior of $${z}_{t}$$, the prior for the next time step $${z}_{t+1}$$ is estimated. Such data assimilation enables real-time estimation of latent states and generation of virtual ECoG signals.

### Parameter setting

The model architecture was initially guided by understanding that the brain engages in hierarchical information processing, with distinct levels: one that governs overall brain states, such as levels of consciousness, another that controls networks through integration of different brain regions, and a third that operates at the level of individual brain regions^59–62^. Based on this understanding, we structured our model into three hierarchical levels: the global state level, the functional network level, and the local region level. At the global state level, we included two latent units, z, as the minimum necessary to represent conditions of wakefulness and individual characteristics. At the functional network level, three latent units, z, were implemented to minimally represent the expected number of latent networks. For the local region level, the number of latent units, z, was set at ten, in reference to previous research^19,61^, assigning one latent dimension per two input signal dimensions, which corresponds to ten latent units for twenty input channels. The number of deterministic units, d, was determined by assessing the minimum required to reduce reconstruction error. The pilot analysis indicated that 15 deterministic units were necessary at the functional network level, while 15 deterministic units per single latent unit were required at the local region level.

During training, the weights, w, and posterior of latent state ${\rm{z}}$ values were updated 200,000 times. This number of updates was chosen based on the stability and consistent decrease in the loss function across different training datasets during cross-validation. In the data assimilation phase, particularly during real-time latent state estimation experiments, the time window was set to 500 time steps. Within this window, forward prediction generation and backpropagation-based updates of the posterior for the latent variable unit ${\rm{z}}$ were repeated 100 times. After each set of updates, the time window was advanced by one time step. Consequently, by the time the window reached the final time step, the posterior for the latent variable unit z at each time step had been updated 500×100 = 50,000 times. Reducing the time window or the number of updates excessively could lead to increased prediction error and weakened clustering between wakefulness and anesthesia, represented by z^(3)^µ (Supplementary Tables 3-6). For the virtual intervention experiment, the time window ${\rm{H}}$ was fixed at 2,000 time steps, and posterior values were updated 10,000 times. The reason for fixing the time window to the sequence length was to ensure that z^(3)^ did not change temporally, allowing it to abstractly represent the characteristics of the entire ECoG sequence. Hyperparameters of the Rectified Adam optimizer during learning and data assimilation stages were set to their default values: α = 0.001 (learning rate), β1 = 0.9, and β2 = 0.999.

### Virtual intervention

Virtual intervention experiments were performed on latent states of z^(3)^, z^(2)^, z^(1)^. In these experiments, we generated virtual ECoGs by inputting latent states estimated as anesthetized or awake test individuals in target latent units. Then, we investigated whether resulting virtual ECoG waveforms exhibited characteristics of anesthetized or awake conditions. Latent states used as intervention inputs were posterior distributions obtained through data assimilation using ECoG signals of test individuals (See also the section of Parameter setting).

In virtual interventions targeting z^(3)^, the posterior of the constant z^(3)^ was input to latent units together with the posterior of z^(2)^ and z^(1)^ to generate virtual ECoGs. Effects of this virtual intervention were evaluated based on the proportion of generated ECoGs indicating anesthesia or awake characteristics. Assessment of these characteristics in ECoG signals utilized a discriminator, as described below.

As part of functional analysis, virtual interventions targeting z^(2)^ and z^(1)^ were conducted. This was undertaken to investigate the contribution of each latent unit in altering generated ECoGs from awake to anesthetized conditions. Initially, the posterior of an awake condition was inputted to z^(3)^ so that awake ECoGs were generated in the absence of intervention. Subsequently, the posterior of anesthesia was individually inputted to specific latent units of z^(2)^ or z^(1)^ (virtual intervention) and virtual ECoGs were generated. The contribution of each latent unit to anesthetized ECoG generation was evaluated based on the proportion of generated ECoG manifesting anesthetic characteristics, which was assessed by the discriminator, as described below. In these analyses, target latent units had their posterior distributions inputted, while non-target latent units underwent top-down prediction generation using prior distributions without explicit setting of distributions corresponding to awake or anesthetized conditions.

### Discriminator

In order to examine characteristics of virtually generated ECoG signals, a discriminator was used. This discriminator made classifications as to whether virtual ECoG signals pertained to states of anesthesia or wakefulness, or in which of the four individuals ECoG signals originated. The discriminator employed a model that converts ECoGs into spectrograms and utilizes a deep convolutional neural network (CNN) for classification, noted in previous studies for its high accuracy^63^. Training for this model utilized ECoG signals across all individuals in the study, during both anesthesia and wakefulness. Generation of spectrograms was carried out by applying the multi-taper time-frequency spectrum method to ECoG sequences that were segmented every 2000ms. During this process, the length of the moving window was set to 100 ms, with a step size of 10 ms, and calculations were performed for the 0-200 Hz frequency band. Spectrogram images across 20 channels were compiled as a single-data unit and subjected to discriminant analysis using a three-dimensional CNN, the structure of which is detailed in Supplementary Fig. 7. In the training phase of this CNN, cross-entropy was employed as the loss function, and the model was updated 100 times using an Adam optimizer with a learning rate of 0.0005. Models were constructed for both binary classification tasks, distinguishing between anesthesia and wakefulness, and for a four-class identification task involving individual differentiation. Test accuracies for these models were remarkably high, at 99.6% and 98.1%, respectively.

### Clustering metrics

Cluster structures emerging in the distribution of latent states z^(3)^ at a global state level were quantitatively assessed. For this assessment, silhouette width^64^, a method commonly used for cluster evaluation, was used for the z^(3)^µ space as shown in Fig. 3a, and the calculation process is shown below. Consider the case where data point $i$ corresponding to one z^(3)^µ state (corresponding to one point in Fig. 3a) is to be evaluated as to whether it belongs to cluster ${C}_{i}$. To begin with, ${\rm{a}}\left(i\right)$ is defined as follows.

17 $${\rm{a}}\left(i\right)=\frac{1}{\left|{C}_{i}\right|-1}\sum _{j\in {C}_{i},i\ne j}d\left(i,j\right)$$

Here, $\left|{C}_{i}\right|$ is the number of points belonging to cluster ${C}_{i}$, and $d\left(i,j\right)$ is the distance between data points $i$ and $j$. ${\rm{a}}\left(i\right)$ represents the average of the distances from data point $i$ to the rest of the points in its cluster ${C}_{i}$, indicating that a reduced ${\rm{a}}\left(i\right)$ value reflects greater cohesion in cluster ${C}_{i}$. Next, ${\rm{b}}\left(i\right)$ is defined as follows.

18 $${\rm{b}}\left(i\right)=\mathop{\min }\limits_{k\ne i}\frac{1}{\left|{C}_{k}\right|}\sum _{j\in {C}_{k}}d\left(i,j\right)$$

Here, ${\rm{b}}\left(i\right)$ represents the average distance between data point $i$ and points to the adjacent, but distinct cluster, indicating that a higher ${\rm{b}}\left(i\right)$ value suggests a greater separation from adjacent clusters.

Based on ${\rm{a}}\left(i\right)$ and ${\rm{b}}\left(i\right)$, the silhouette width ${\rm{s}}\left(i\right)$ for data point $i$ is calculated as follows.

19 $${\rm{s}}\left(i\right)=\frac{b\left(i\right)-a\left(i\right)}{\max \left\{a\left(i\right),b\left(i\right)\right\}}$$

A higher value of s(i) indicates that data point $i$ is more strongly clustered in ${C}_{i}$. In real-time latent state estimation experiments, we utilized ${\rm{s}}\left(i\right)$ to classify the z^(3)^µ of each time step, obtained through data assimilation, as belonging to either the anesthesia or awake clusters established during the learning process. Specifically, ${\rm{s}}\left(i\right)$ was calculated for both the anesthesia and awake clusters, and data point $i$ was classified into the cluster with larger ${\rm{s}}\left(i\right)$.

From Eq. (19), it can be inferred that if ${\rm{s}}\left(i\right)\,$> 0, data point $i$ tends to be clustered into ${C}_{i}$, rather than being randomly distributed. In this study, we utilized this principle to investigate whether individual clusters emerge in the z^(3)^ space. Specifically, the individual to which each data point $i$ belongs was designated as ${C}_{i}$ and ${\rm{s}}\left(i\right)$ was calculated for all data points in the training ECoG sequences to statistically examine whether the average silhouette width exceeds zero (t-test).

To determine whether the position of z^(3)^ obtained from data assimilation belongs to the anesthesia cluster or the wakefulness cluster identified during training, we used k-Nearest Neighbors (k-NN). The hyperparameter (k-value) for k-NN was determined through leave-one-out cross-validation in the training data.

Finally, to ensure transparent reporting, we included the TRIPOD checklist as Supplementary Fig. 8.

## Data availablity

ECoG data utilized in this study are available in neurotycho, http://www.neurotycho.org/.

## Supplementary information

Supplementary Information

Supplementary Movie 1

## Data Availability

The code for the V-RNN, a principal component of the model discussed herein, is shared at: https://github.com/h-idei/pvrnn_sa.
