# Supplementary material for: Digital twin brain simulator for real-time consciousness monitoring and virtual intervention using primate electrocorticogram data
Source: NPJ Digit Med. 2025 Feb 10;8:80. doi: 10.1038/s41746-025-01444-1 (PMC11811282; doi:10.1038/s41746-025-01444-1)
Supplement: Supplementary file 1 — Supplementary Information [file 41746_2025_1444_MOESM1_ESM.pdf]

Supplementary Information

Supplementary Figure 1: Learning curve.

Supplementary Figure 2: Power spectrum density of ECoG signals depending on the brain regions.

Supplementary Figure 3: Power spectrum density of ECoG signals depending on the individuals.

Supplementary Figure 4: Evaluation of the similarity in functional connectivity between observed and predicted ECoG signals.

Supplementary Figure 5: Temporal processing of the V-RNN.

Supplementary Figure 6: Data assimilation phase

Supplementary Figure 7: Network architecture of discriminator.

Supplementary Figure 8: Transparent reporting of a multivariable prediction model for individual prognosis or diagnosis (TRIPOD) Checklist.

Supplementary Table 1: The proportion of correct cluster localization based on the silhouette width matric.

Supplementary Table 2: Prediction error when predicting the future ECoG signals during prior generation.

Supplementary Table 3: Prediction error depending on the number of update times.

Supplementary Table 4: The proportion of $z^{(3)}$s in the correct cluster depending on the number of update times.

Supplementary Table 5: Prediction error depending on the widths of the time windows.

Supplementary Table 6: The proportion of $z^{(3)}$s in the correct cluster depending on the widths of the time windows.

Supplementary Movie 1: Real-time latent state estimation via data assimilation.

Supplementary Figure 1. Learning curve.

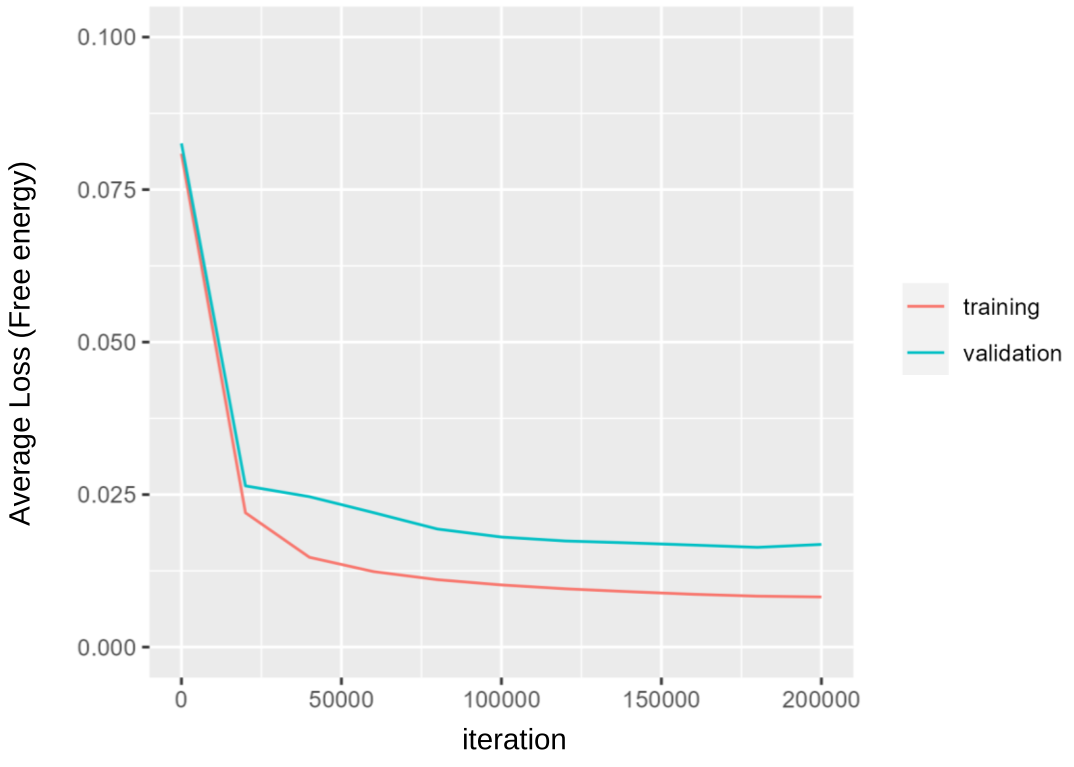

This figure presents the average values of the loss function during 4-fold cross-validation for the model in the course of training. The red and cyan lines represent the results calculated using the training and validation data, respectively. The training data consists of the 72 sequences described in the Methods section. For the validation data, 2000 time step ECoG sequences, independent of the training data, were used, similar to the virtual drug intervention experiments.

Supplementary Figure 2. Power spectrum density of ECoG signals depending on the brain regions.

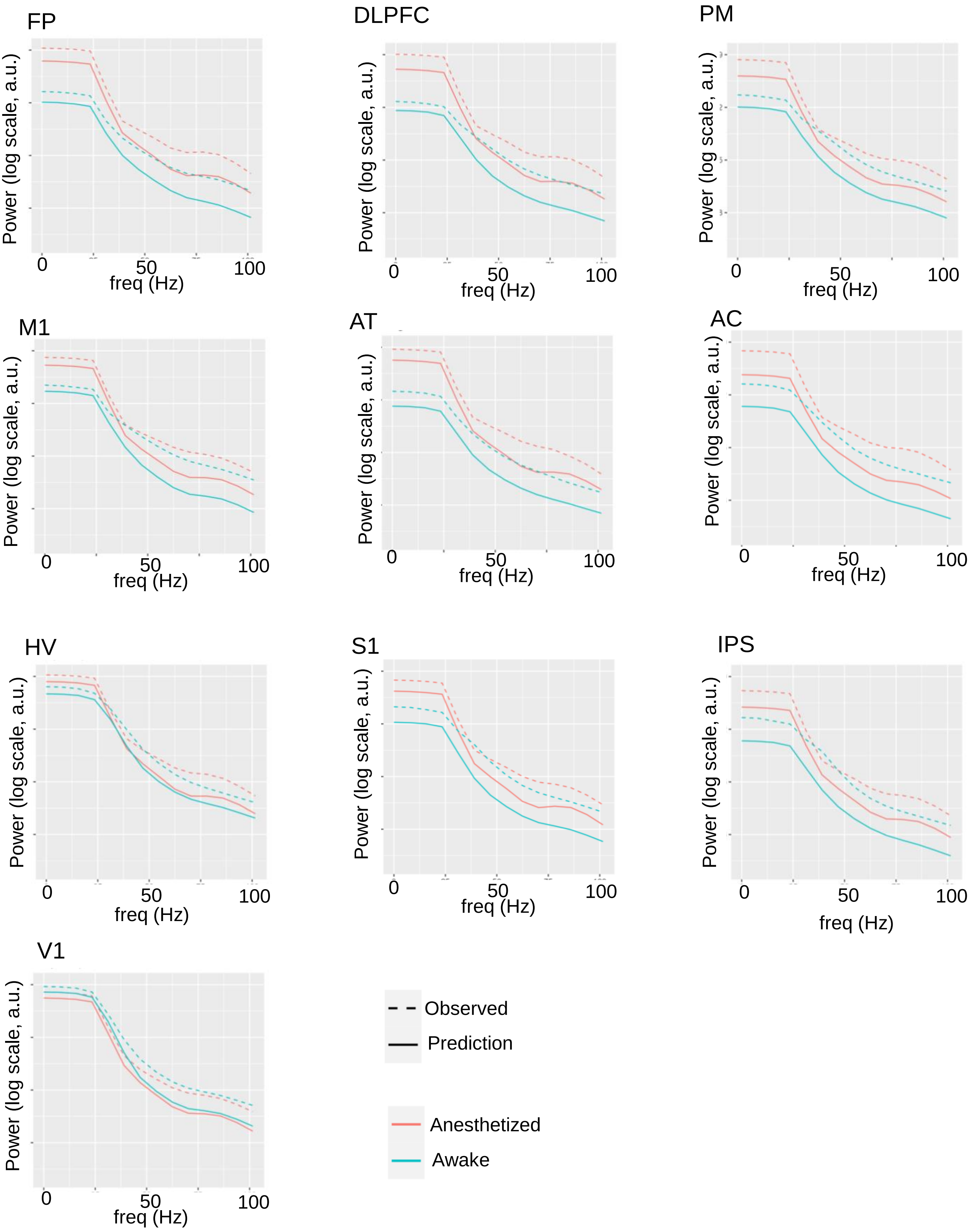

The power spectrum was generated by applying the multi-taper time-frequency spectrum method to ECoG sequences segmented into 2000ms intervals, and was calculated by averaging across time steps, sequences, channels, and cross-validated networks. Abbreviations: FP, frontal pole; DLPFC, dorsolateral prefrontal cortex; PM, premortor cortex; M1, primary motor cortex; S1, primary somatosensory cortex; IPS, intraparietal sulcus; AT, anterior temporal cortex; AC, auditory cortex; HV, higher visual cortex; V1, primary visual cortex.

Supplementary Figure 3. Power spectrum density of ECoG signals depending on the individuals.

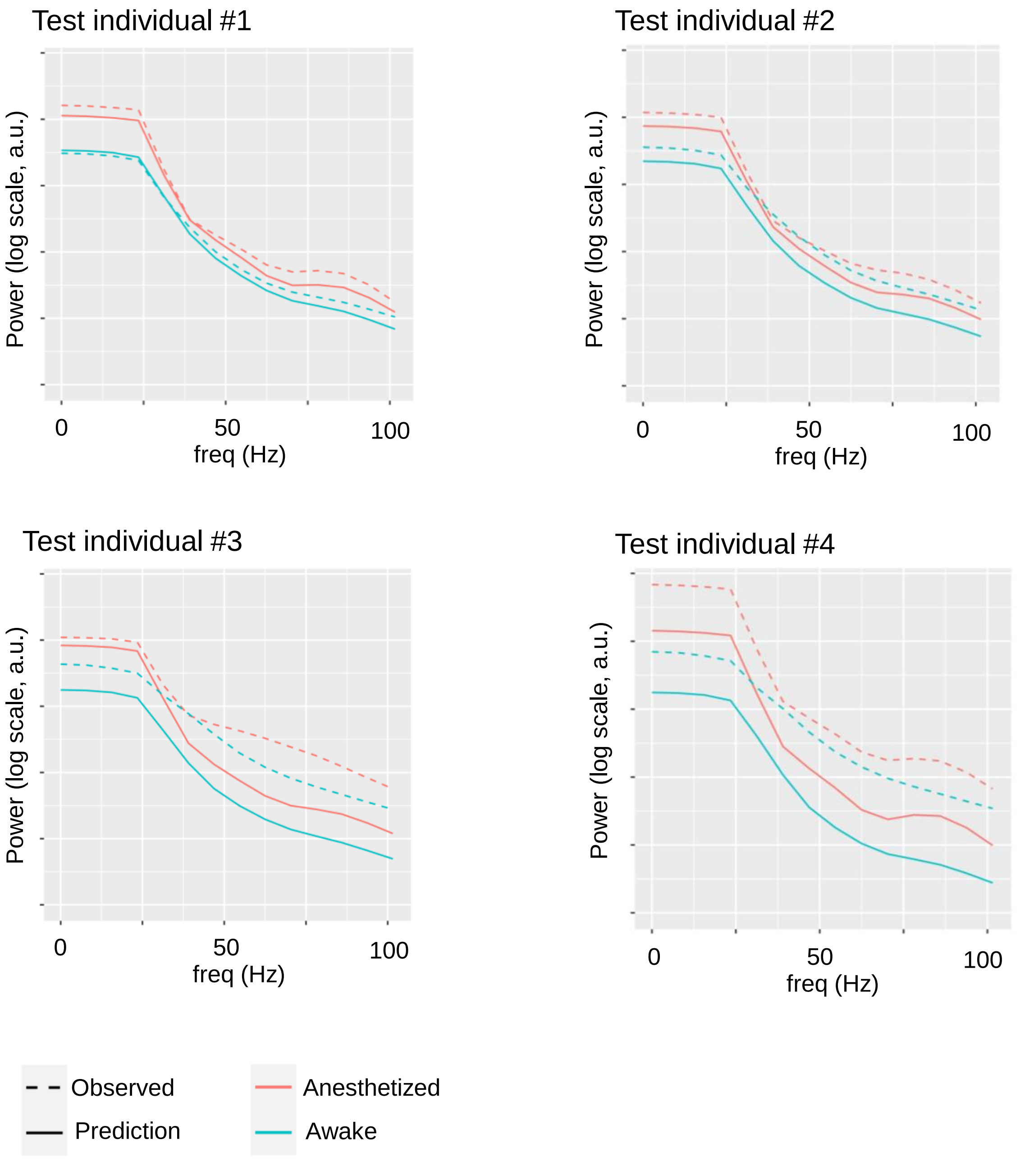

The power spectrum was generated by applying the multi-taper time-frequency spectrum method to ECoG sequences segmented into 2000ms intervals, and was calculated by averaging across time steps, sequences, and channels.

Supplementary Figure 4. Evaluation of the similarity in functional connectivity between observed and predicted ECoG signals.

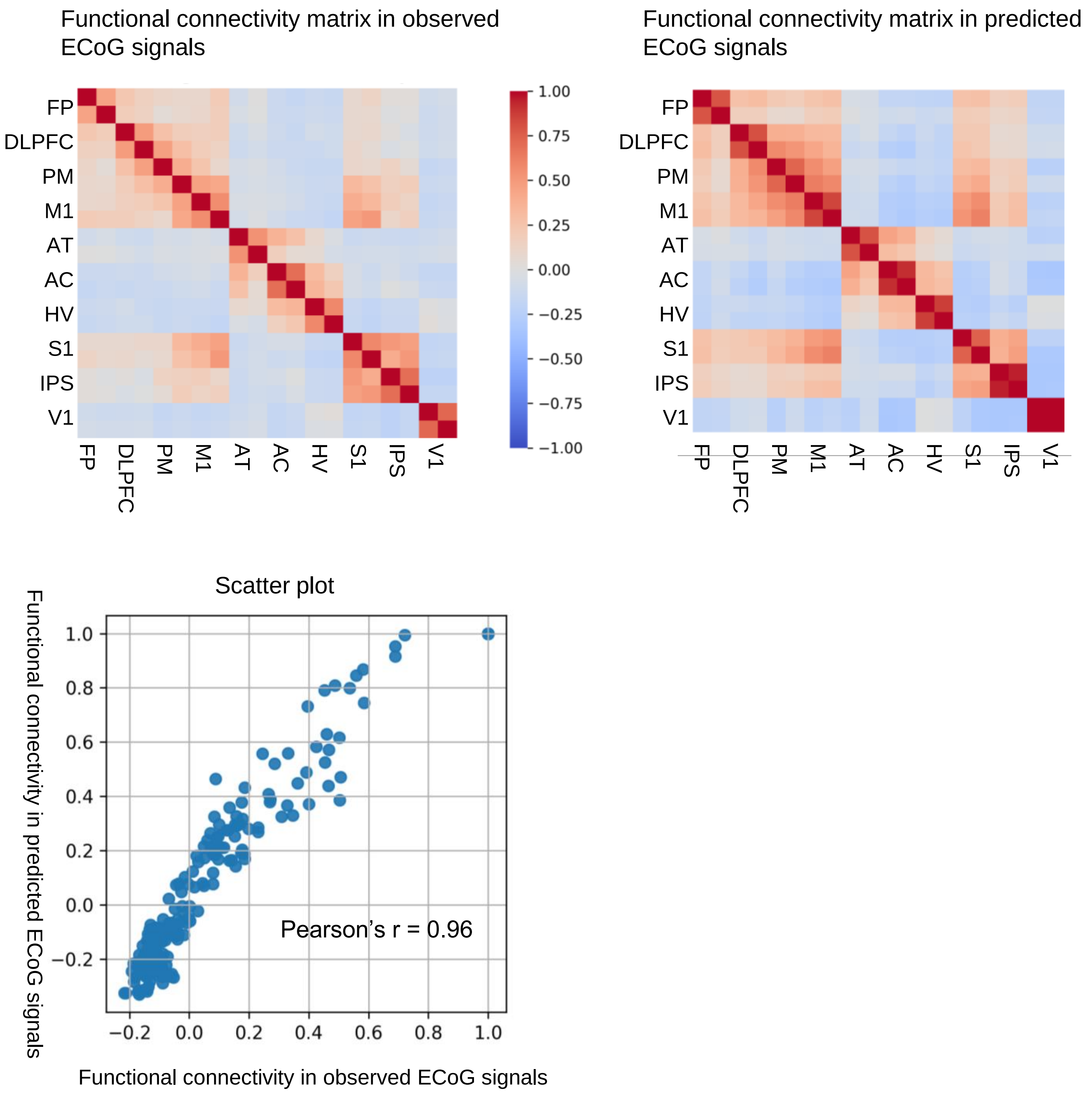

To examine the similarity in functional connectivity between observed ECoG signals from test individuals and ECoG signals predicted through data assimilation, functional connectivity matrices were constructed. The test data used consisted of 100 randomly selected ECoG signal segments of 2,000 time steps each (50 anesthetized and 50 awake), derived from the test individual's ECoG signals. These are the same data used for the virtual intervention experiments on $z^{(3)}$. For further details, refer to the "Data and preprocessing" section in the Methods. Abbreviations: FP, frontal pole; DLPFC, dorsolateral prefrontal cortex; PM, premortor cortex; M1, primary motor cortex; S1, primary somatosensory cortex; IPS, intraparietal sulcus; AT, anterior temporal cortex; AC, auditory cortex; HV, higher visual cortex; V1, primary visual cortex.

Supplementary Figure 5. Temporal processing of the V-RNN.

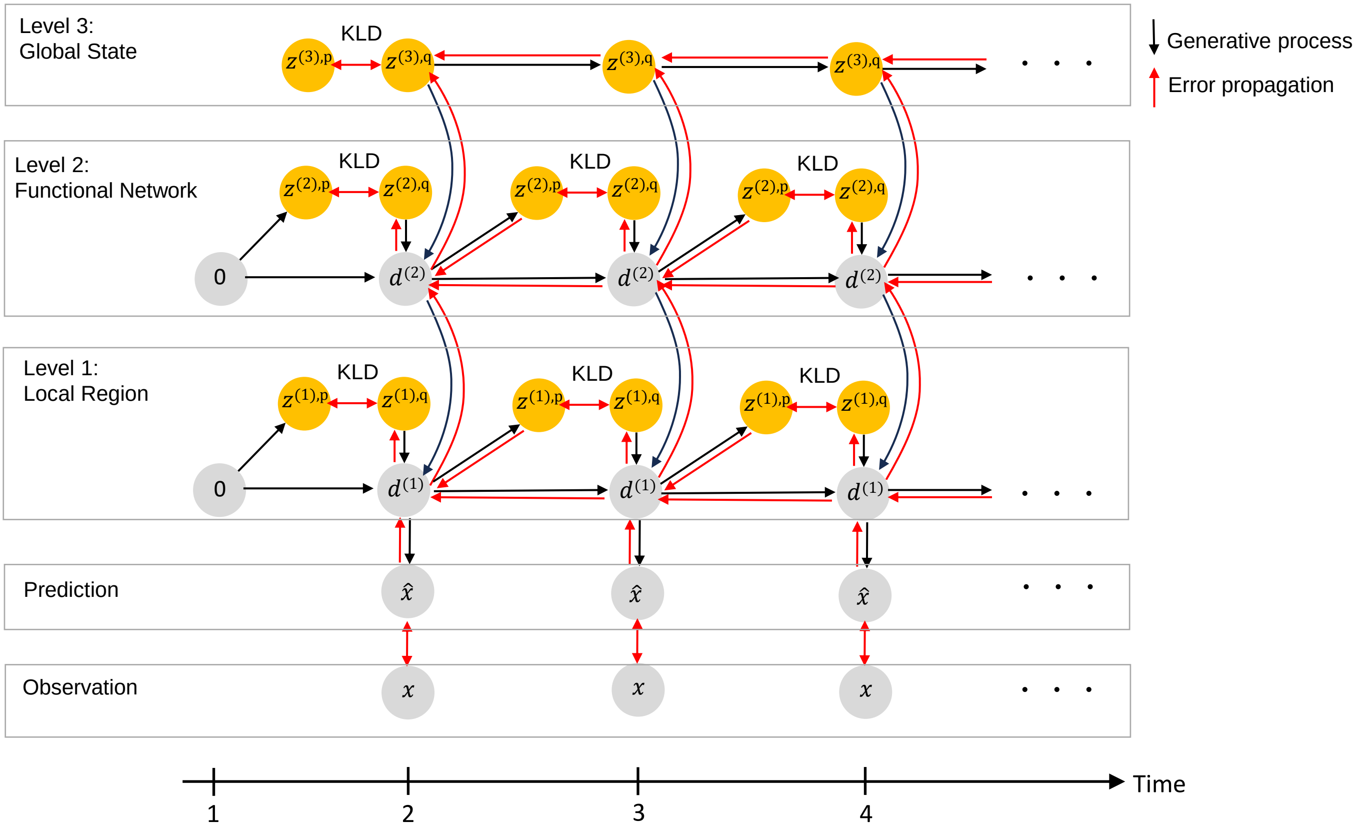

During the training phase, the network optimizes the posteriors of latent variables $z_{1:T}$ across all modules and updates time-constant synaptic weights by minimizing the free energy F over the duration T of training ECoG sequences. Initial deterministic states of all modules are set to zero. For simplicity, only one out of ten modules is depicted for local region modules. Abbreviation: KLD, Kullback-Leibler divergence.

Supplementary Figure 6. Data assimilation phase.

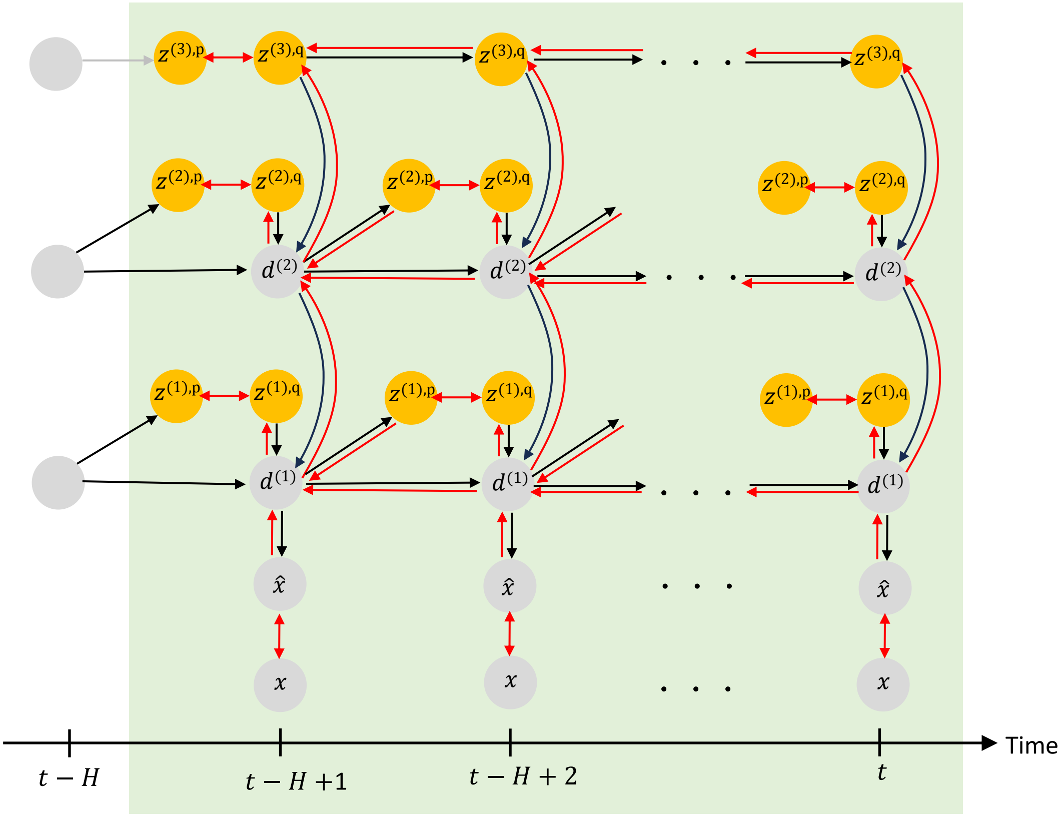

Data assimilation encompasses a cyclical process that integrates top-down predictions with bottom-up posterior updates driven by observations. This iterative procedure is conducted in a sliding time window that retraces H time steps from the current time step t, progressively moving forward as time advances. During this phase, synaptic weights remain fixed.

Supplementary Figure 7. Network architecture of discriminator.

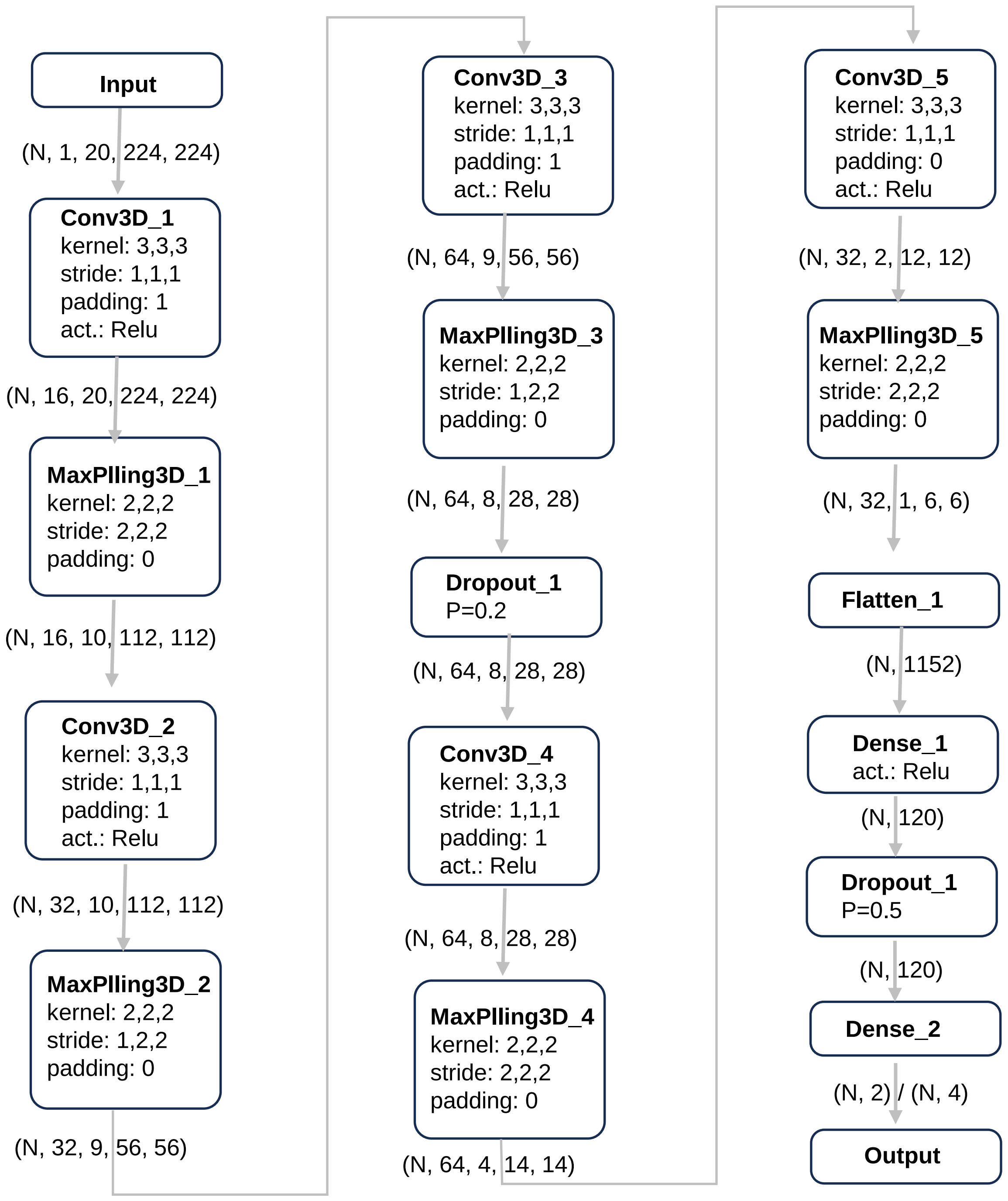

In the Three-Dimensional Convolutional Neural Network (3DCNN) architecture, Conv3D refers to the 3D convolution layer that extracts features from spatial and temporal data. MaxPooling3D is the 3D max pooling layer, which reduces the size of the data by selecting the maximum value in each region. ReLU, or Rectified Linear Unit, is an activation function that outputs the input if it's positive and zero if negative. Dropout is a regularization technique that randomly drops neurons during training to prevent overfitting and improve generalization.

Supplementary Figure 8. Transparent reporting of a multivariable prediction model for individual prognosis or diagnosis (TRIPOD) Checklist.

TRIPOD Checklist: Prediction Model Development

Section/Topic	Item	Checklist Item	Page
**Title and abstract**			
Title	1	Identify the study as developing and/or validating a multivariable prediction model, the target population, and the outcome to be predicted.	1
Abstract	2	Provide a summary of objectives, study design, setting, participants, sample size, predictors, outcome, statistical analysis, results, and conclusions.	3
**Introduction**			
Background and objectives	3a	Explain the medical context (including whether diagnostic or prognostic) and rationale for developing or validating the multivariable prediction model, including references to existing models.	4-6
	3b	Specify the objectives, including whether the study describes the development or validation of the model or both.	6-7
**Methods**			
Source of data	4a	Describe the study design or source of data (e.g., randomized trial, cohort, or registry data), separately for the development and validation data sets, if applicable.	25
	4b	Specify the key study dates, including start of accrual; end of accrual; and, if applicable, end of follow-up.	N/A
Participants	5a	Specify key elements of the study setting (e.g., primary care, secondary care, general population) including number and location of centres.	N/A
	5b	Describe eligibility criteria for participants.	25
	5c	Give details of treatments received, if relevant.	N/A
Outcome	6a	Clearly define the outcome that is predicted by the prediction model, including how and when assessed.	36-38
	6b	Report any actions to blind assessment of the outcome to be predicted.	36-38
Predictors	7a	Clearly define all predictors used in developing or validating the multivariable prediction model, including how and when they were measured.	28-31
	7b	Report any actions to blind assessment of predictors for the outcome and other predictors.	N/A
Sample size	8	Explain how the study size was arrived at.	8, 25
Missing data	9	Describe how missing data were handled (e.g., complete-case analysis, single imputation, multiple imputation) with details of any imputation method.	N/A
Statistical analysis methods	10a	Describe how predictors were handled in the analyses.	28-31
	10b	Specify type of model, all model-building procedures (including any predictor selection), and method for internal validation.	27-28
	10d	Specify all measures used to assess model performance and, if relevant, to compare multiple models.	36-38
Risk groups	11	Provide details on how risk groups were created, if done.	N/A
**Results**			
Participants	13a	Describe the flow of participants through the study, including the number of participants with and without the outcome and, if applicable, a summary of the follow-up time. A diagram may be helpful.	8
	13b	Describe the characteristics of the participants (basic demographics, clinical features, available predictors), including the number of participants with missing data for predictors and outcome.	8
Model development	14a	Specify the number of participants and outcome events in each analysis.	8
	14b	If done, report the unadjusted association between each candidate predictor and outcome.	N/A
Model specification	15a	Present the full prediction model to allow predictions for individuals (i.e., all regression coefficients, and model intercept or baseline survival at a given time point).	8-9
	15b	Explain how to the use the prediction model.	8-9
Model performance	16	Report performance measures (with CIs) for the prediction model.	9-11
**Discussion**			
Limitations	18	Discuss any limitations of the study (such as nonrepresentative sample, few events per predictor, missing data).	21-23
Interpretation	19b	Give an overall interpretation of the results, considering objectives, limitations, and results from similar studies, and other relevant evidence.	16-20
Implications	20	Discuss the potential clinical use of the model and implications for future research.	24
**Other information**			
Supplementary information	21	Provide information about the availability of supplementary resources, such as study protocol, Web calculator, and data sets.	39
Funding	22	Give the source of funding and the role of the funders for the present study.	39

We completed the TRIPOD checklist to enhance the transparency and completeness of reporting in studies developing prediction models.

Supplementary Table 1. The proportion of correct cluster localization based on the silhouette width matric.

	Before the switch	After the switch
Mean	0.69	0.62
SD	0.19	0.15

Using the silhouette width matric (Methods section for details), we evaluated whether the $z^{(3)}$ states during data assimilation were appropriately positioned in clusters of wakefulness or anesthesia formed during training

Supplementary Table 2. Prediction error when predicting the future ECoG signals during prior generation.

The predicted future time step	1 step	5 step	10 step	20 step	50 step	100 step
Mean	1.1×10^{-3}	1.1×10^{-3}	1.2×10^{-3}	1.3×10^{-3}	1.7×10^{-3}	1.9×10^{-3}
SD	1.2×10^{-3}	1.2×10^{-3}	1.2×10^{-3}	1.3×10^{-3}	1.5×10^{-3}	1.7×10^{-3}

This table shows the prediction error during data assimilation when predicting future time steps from the head of the time window's latent states using prior generation.

Supplementary Table 3. Prediction error depending on the number of update times.

The number of update times	100	50	25	10
Mean	7.0×10^{-4}	7.6×10^{-4}	9.3×10^{-4}	1.4×10^{-3}
SD	9.2×10^{-4}	9.9×10^{-4}	1.1×10^{-3}	1.5×10^{-3}

In real-time latent state estimation, the main results were obtained by setting the number of update times in data assimilation to 100. This table shows the changes in prediction error when the number of update times is reduced.

Supplementary Table 4. The proportion of $z^{(3)}$s in the correct cluster depending on the number of update times.

The number of update times		100	50	25	10
Before condition switch	Mean	0.78	0.77	0.76	0.61
	SD	0.13	0.16	0.16	0.08
After condition switch	Mean	0.67	0.66	0.60	0.51
	SD	0.13	0.15	0.12	0.06

During the real-time latent state estimation, the proportion of inferred $z^{(3)}$ values that were located within the correct cluster (either anesthesia or wakefulness, as identified during training) was computed. The clustering metric used for this analysis was k-Nearest Neighbors (detailed in Methods).

Supplementary Table 5. Prediction error depending on the widths of the time windows.

Window width	500	250	100	50	25
Mean	7.0×10^{-4}	6.8×10^{-4}	7.1×10^{-4}	8.1×10^{-4}	1.1×10^{-3}
SD	9.2×10^{-4}	8.7×10^{-4}	9.0×10^{-4}	9.7×10^{-4}	1.2×10^{-3}

In real-time latent state estimation, the main results were obtained by setting the window width in data assimilation to 500. This table shows the changes in prediction error when the window width is reduced.

Supplementary Table 6. The proportion of $z^{(3)}$s in the correct cluster depending on the widths of the time windows.

Window width		500	250	100	50	25
Before condition switch	Mean	0.78	0.78	0.79	0.77	0.67
	SD	0.13	0.11	0.04	0.04	0.03
After condition switch	Mean	0.67	0.66	0.64	0.59	0.56
	SD	0.13	0.11	0.16	0.14	0.06

During the real-time latent state estimation, the proportion of inferred $z^{(3)}$ values that were located within the correct cluster (either anesthesia or wakefulness, as identified during training) was computed. The clustering metric used for this analysis was k-Nearest Neighbors (detailed in Methods).

Supplementary Movie 1. Real-time latent state estimation via data assimilation.

Prediction and latent state sequences in data assimilation. This movie illustrates the sequence of predictions and the mean (μ) of posterior distributions of latent states $z^{(1)}$, $z^{(2)}$, $z^{(3)}$, generated in real-time in response to observed electrocorticogram (ECoG) data. The Observed ECoG combines 2500 time steps (2.5 seconds) of awake ECoG observations followed by 1500 time steps (1.5 seconds) of anesthetized ECoG observations from a test individual (an individual not included in the training dataset). The black rectangle indicates the time window during which data assimilation is performed. Within this window, the posterior of the latent states is updated based on the prediction error (PE), which is the difference between observed and predicted ECoG, and new predictions are made based on the updated posterior. Predicted and observed ECoG signals are visualized by selecting 5 channels from the original 20 channels for visual clarity. Specifically, one channel is chosen from each of the frontal pole (FP), primary motor cortex (M1), primary somatosensory cortex (S1), intraparietal sulcus (IPS), and primary visual cortex (V1) regions. Similarly, the $z^{(1)}\mu$ sequences were also visualized by selecting these five regions.
